# Antiproliferative Activity, Multikinase Inhibition, Apoptosis- Inducing Effects and Molecular Docking of Novel Isatin–Purine Hybrids

**DOI:** 10.3390/medicina59030610

**Published:** 2023-03-19

**Authors:** Ashwag S. Alanazi, Tebyan O. Mirgany, Aisha A. Alsfouk, Nawaf A. Alsaif, Mohammed M. Alanazi

**Affiliations:** 1 Department of Pharmaceutical Sciences, College of Pharmacy, Princess Nourah bint Abdulrahman University, Riyadh 84428, Saudi Arabia; 2 Department of Pharmaceutical Chemistry, College of Pharmacy, King Saud University, Riyadh 11451, Saudi Arabia

**Keywords:** anticancer, purine, isatin, hybrid design, kinase inhibitor

## Abstract

The traditional single-treatment strategy for cancer is frequently unsuccessful due to the complexity of cellular signaling. However, suppression of multiple targets is vital to defeat tumor cells. In this research, new compounds for the treatment of cancer were developed successfully as novel hybrid anticancer agents. Based on a molecular hybridization strategy, we designed hybrid agents that target multiple protein kinases to fight cancer cells. The proposed hybrid agents combined purine and isatin moieties in their structures with 4-aminobenzohydrazide and hydrazine as different linkers. Having those two moieties in one molecule enabled the capability to inhibit multiple kinases, such as human epidermal receptor (EGFR), human epidermal growth factor receptor 2 (HER2), vascular endothelial growth factor receptor 2 (VEGFR2) and cyclin-dependent kinase 2 (CDK2). Anticancer activity was evaluated by performing cytotoxicity assays, kinase inhibition assays, cell cycle analysis, and BAX, Bcl-2, Caspase 3 and Caspase 9 protein level determination assays. The results showed that the designed hybrids tackled the cancer by inhibiting both cell proliferation and metastasis. A molecular docking study was performed to predict possible binding interactions in the active site of the investigated protein kinase enzymes.

## 1. Introduction

Cancer is one of the most serious diseases, and the second leading cause of death. It is described by overexcited and uncontrolled cell proliferation [1]. Around one third of the population worldwide is expected to be diagnosed with cancer during their lifetimes [2]. According to the International Agency for Research on Cancer, in 2018 about 18.1 million people were diagnosed with cancer and around 9.6 million people died of the disease. It is estimated that the morbidity and mortality of cancer will continue to rise, with cancer cases growing to 24.1 million new cases and 13.0 million deaths by 2030 [3]. When an appropriate treatment strategy is provided, around two thirds of human cancers can be treated effectively [4]. The development of treatment resistance is the major obstacle to successful cancer therapy. The most effective therapies frequently fail to result in a thorough tumor response, and eventually end up with treatment resistance and tumor deterioration [5]. Therefore, continuous exploration of novel anticancer agents with low toxicity and high efficiency against both drug-sensitive and drug-resistant cancers are still imperative goals for the research and development of anticancer drugs [6]. Additionally, treating cancer patients with more than one drug has a number of negative consequences. These consequences can be avoided by using a single drug with multiple molecular targets, which nowadays is a much-preferred therapeutic strategy [7,8,9].

PTKs (protein tyrosine kinases) are crucial enzymes in cell proliferation [10]. PTKs phosphorylate amino acid residues in proteins, inducing a folding change in the proteins’ 3D structure that ultimately affects protein function [11]. PTKs have a critical role in the development and progression of cancer. PTK dysfunction is implicated in most cancer disorders [1]. Furthermore, PTKs produce more than 60% of all key oncoproteins and proto-oncoproteins in cancer disease [11]. Therefore, PTKs are a good target for cancer therapy for medicinal chemists and biologists.

Human epidermal growth factor receptors (HERs), also known as HER/ErbB, are a tyrosine kinase transmembrane receptor family that includes members such as EGFR (HER1), HER2, HER3, and HER4 [12]. These receptors have three domains: an extracellular ligand binding domain, a transmembrane domain, and an intracellular tyrosine kinase domain. The tyrosine kinase receptors are key proteins in cancer development, including cell overexpression and inadequate suppression in a variety of human tumor cells [5]. Abnormal HER family signaling facilitates invasion, metastasis, angiogenesis, and resistance to apoptosis. Many cancer cells, including ovarian, prostate, colon, and breast cancer cells, have been linked to overexpressed EGFR and HER2 receptors. Therefore, inhibiting the overexpression of these receptors leads to apoptosis in a variety of solid tumors, including breast and lung cancer [13] tumors.

Cancer angiogenesis is a fundamental step for tumor growth because it assures the supply of oxygen and nutrients to proliferating cells by forming new blood vessels, which can lead to cancer progression and metastasis [14]. Vascular endothelial growth factor receptors (VEGFRs) are also PTKs, and have been identified as an outstanding therapeutic target for developing new anticancer agents [15,16]. Among the class of VEGFRs, VEGFR-2 has a critical role in tumor angiogenesis. The expression of VEGFR-2 in endothelial cells of the tumor vasculature is much greater than its expression in normal endothelial cells [17]. Overexpressed VEGFR-2 is found in a variety of tumors, including hepatocellular carcinoma and breast cancer. Thus, EGFR, HER2 and VEGFR2 are important targets for anticancer drug design and development [18].

**Figure 1:**
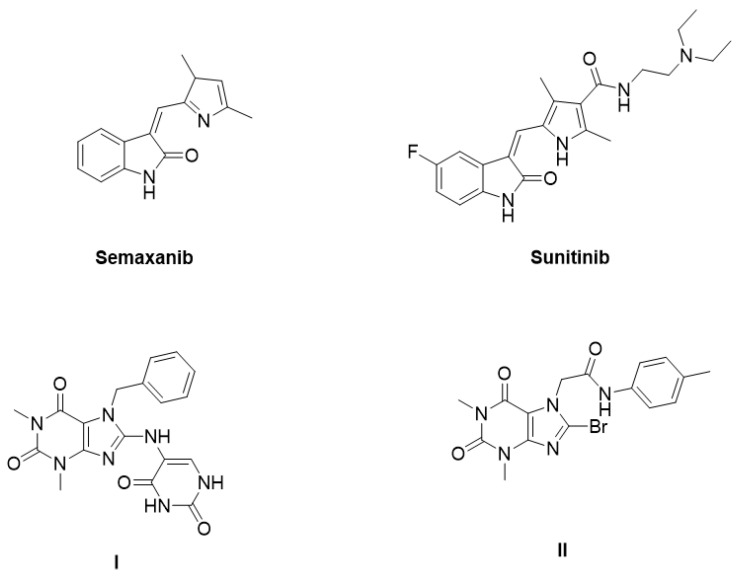
Compounds containing isatin scaffold or purine.

Isatin-bearing (indole-2,3-dione) compounds have a broad spectrum of pharmacological activities, including antibacterial, antimalarial, antitubercular, and anticancer activities. Isatin derivatives have the potential to modulate multiple biological targets, such as histone deacetylase, β-carbonic anhydrase, tyrosine kinase, phosphodiesterase 4B, and tubulin, changing the expression of particular apoptosis-related genes and eventually causing apoptosis [19]. Furthermore, several potent antitumor agents, such as semaxanib and sunitinib, contain an isatin scaffold (Figure 1), demonstrating that isatin is an effective moiety for the development of novel anticancer agents [20]. On the other hand, purine-containing compounds such as **I** and **II** (Figure 1) were also found to be good anticancer agents due to their multitarget effects, including the inhibition of the activity of CDKs, EGFR, VEGFR2, HDAC, mTOR, MAPK and MNK [21,22,23,24,25,26,27].

### Aim of the Study

**Figure 2:**
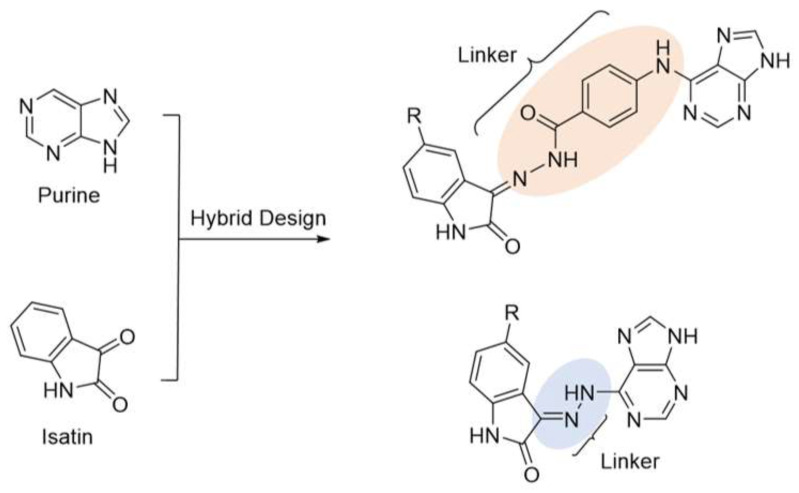
Rational design of isatin–purine hybrid compounds.

Each cell represents various genetic makeups, due to differences in the active oncogenes and inactive tumor suppressor genes. A single drug may successfully destroy cells holding a specific alteration while cells driven by other stimulants persist [28]. The complexity of cellular signaling frequently makes the traditional single-treatment strategy unsuccessful, and suppression of multiple targets is vital for defeating tumor cells [29,30]. Therefore, the aim of this study is to develop new drugs with inhibiting effects on cancer cell overexpression. Hybrid compounds were designed (Figure 2) that combine distinct parts from two anticancer agents: isatin-bearing (indole-2,3-dione) compounds and purine-containing compounds. Both parts have been used with FDA-approved drugs. Indeed, isatin–purine hybrid molecules may have the potential to suppress tumor activity, and therefore may provide novel anticancer agents with several molecular targets.

## 2. Results and Discussion

### 2.1. Chemistry

In the design of isatin–purine hybrid compounds **4**–**9**, isatin and purine were linked using 4-aminobenzohydrazide as a linker, while isatin–purine hybrid compounds **11**–**16** were linked using hydrazine as a linker.

**Scheme 1:**
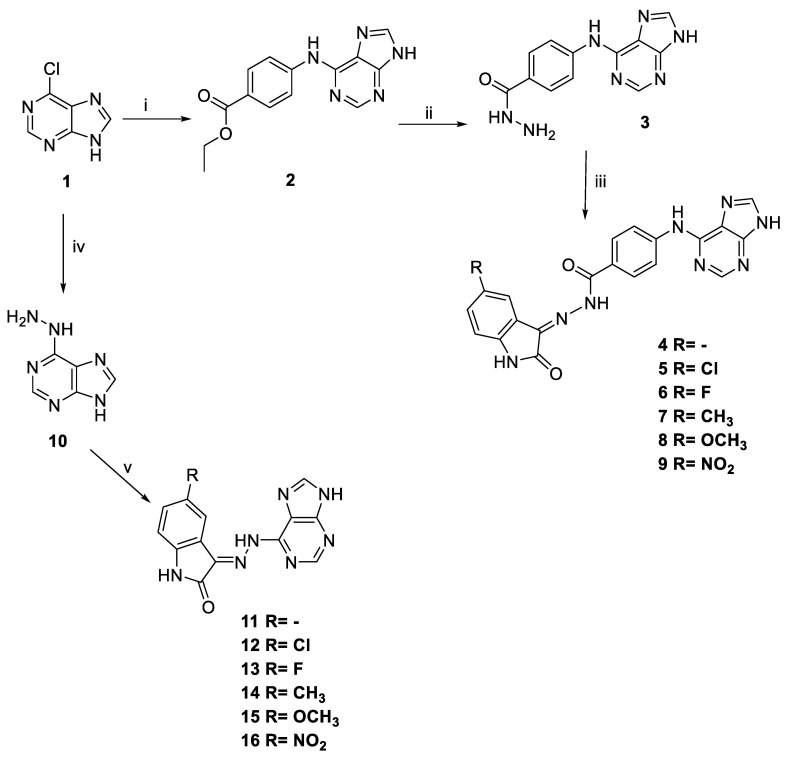
Synthesis of isatin–purine hybrid compounds **4**–**16**. i. Ethyl 4-aminobenzoate, trimethylamine, BuOH, reflux, 7 h. ii. N_2_H_2_.H_2_O, abs. EtOH, reflux, 6 h. iii. Appropriate isatin, AcOH, EtOH, reflux, 8–12 h. iv. Excess of hydrazine hydrate, reflux, 1 h. v. Appropriate isatin, AcOH, EtOH, reflux, 3–7 h.

First, ethyl 4-((9H-purin-6-yl)amino)benzoate 2 (Scheme 1) was obtained from a reaction of ethyl 4-aminobenzoate with a solution of 6-chloro-9*H*-purine in absolute ethanol. The reaction mixture was stirred during reflux for 7 h and the desired product was filtered and washed with cold water. The intermediate (2) was obtained with a yield of 97.38% and fully characterized by ^1^H NMR and LCMS to be used for the next step.

Next, ethyl 4-((9*H*-purin-6-yl)amino)benzoate 2 (Scheme 1) was refluxed in an excess of hydrazine hydrate for 3 h. The mixture was cooled and poured into ice-cold water. The resultant precipitate was filtered, washed thoroughly with water and dried to obtain the desired product, 4-((9*H*-purin-6-yl)amino)benzohydrazide 3. The intermediate 3 was obtained with a yield of 84.15% and fully characterized by ^1^H NMR and LCMS to be used for the next step. 

Synthesis of the final compounds **4**–**9** (Scheme 1) started with mixing 4-((9*H*-purin-6-yl)amino)benzohydrazide 3, the appropriate isatin, and glacial acetic acid in absolute ethanol and refluxing for about 8–12 h. The reaction mixture was cooled and added to ice water, and the formed precipitate was filtered off, washed with water and recrystallized from the proper solvent to obtain the desired compounds. The final compounds were obtained with a yield of 70.72 to 97.08% and fully characterized by ^1^H NMR and LCMS. 

For the second series, 6-hydrazinyl-9H-purine 10 was synthesized first (Scheme 1) by refluxing 6-chloro-9*H*-purine in an excess of hydrazine hydrate for 1 h. The mixture was cooled and poured into ice cold water. The resultant precipitate was filtered, washed thoroughly with water and dried to obtain the intermediate 10. The intermediate 10 was obtained with yield 80.44% and fully characterized by ^1^H NMR and LCMS to be used for the next step.

Compounds **11**–**16** (Scheme 1) were synthesized by following the same procedure as for compounds **4**–**9**, using 6-hydrazinyl-9H-purine 10, the appropriate isatin, and glacial acetic acid in absolute ethanol. The reaction was refluxed for about 3–7 h, then cooled and added to ice water. The formed precipitate was filtered off, washed with water and recrystallized from the proper solvent to obtain the desired compounds. The final compounds were obtained with a yield of 72.46 to 99.5% and fully characterized by ^1^H NMR and LCMS (Supplementary Data).

### 2.2. Biological Evaluation

#### 2.2.1. Cytotoxicity Assay

The in vitro antiproliferative activity of the synthesized hybrid compounds **4**–**9** (isatin–purine hybrid compounds with 4-aminobenzohydrazide spacer) and **11**–**16** (isatin–purine hybrid compounds with hydrazine spacer) was preliminarily assessed using an MTT colorimetric assay against four human cancer cell lines: hepatocellular carcinoma (HepG2), mammary gland cancer (MCF-7), breast cancer (MDA-MB-231) and epithelioid cervix carcinoma (HeLa). The cytotoxic activities of the evaluated compounds are expressed as IC_50_ values (µM), as shown in (Table 1). Sunitinib, a well-known multi-kinase inhibitor, was used as a reference compound. The tested compounds showed varying degrees of antiproliferative activities against the examined cell lines compared to the reference compound. In general, compounds **11**–**16** showed better cytotoxic activity profiles (IC_50_ ≥ 8.93 ± 0.8) than compounds **4**–**9** (IC_50_ ≥ 19.05 ± 1.4 μM) against all tested cancer cell lines except for compound **6** (R=F), which demonstrated better activity than the corresponding compound in the second series (compounds **11**–**16**). Compounds **6**, **8**, **12**, **13**, **14** and **15** demonstrated good antiproliferative activity against all investigated cell lines, with IC_50_ values ranging from 18.06 to 53.49 μM. Intestinally, Compound **15** (R = methoxy) was very potent and displayed highly promising cytotoxic activity against all tested cell lines, with IC_50_ values ranging from 8.93 to 14.89 μM. Additionally, compound **15** demonstrated cytotoxic activity comparable to the reference drug sunitinib against the HepG2, MCF-7, MDA-MB-231 and HeLa cell lines, with IC_50_ values of 9.61, 10.78, 14.89 and 8.93 μM, respectively. On the other hand, the remaining compounds revealed only modest inhibitory activities against the tested cell lines.

#### 2.2.2. Structure–Activity Relationship (SAR)

The structure–activity relationships of the tested isatin–purine hybrid compounds towards HepG2, MCF-7, MDA-MB-231 and HeLa cell lines revealed that most of the compounds **11**–**16** with a hydrazine linker showed more potent cytotoxic activity against all tested cell lines than compounds **4**–**9** with a 4-aminobenzohydrazide linker. This may be because the shorter linker gives compounds better fit and interaction with the targeted kinases. However, compound **6**, with 4-aminobenzohydrazide and R=F, showed more potent activity against the MDA-MB-231 cell line than compound **13**, which has the same R substitution and hydrazine. However, introducing a F group into both 4-aminobenzohydrazide and hydrazine compounds led to better cytotoxicity compared to most other compounds in each series. This may be because the F group was able to form a hydrogen bond during the interaction with the receptors. 

In addition, compound **15**, with a hydrazine linker and R=OCH_3_, was the most potent cytotoxic agent against all tested cancer cell lines. The potency of compound **15** may be due to the fact that hydrazine is a short linker and the methoxy group was able to form a hydrogen bond with the targeted site in the kinase enzymes. 

#### 2.2.3. In Vitro Kinase Inhibitory Activity

The newly prepared compound **15** was further assayed for its inhibitory activity against EGFR, HER2, VEGFR2 and CDK2. The results were reported as 50% inhibition concentration values (IC_50_, expressed as μM) in comparison to erlotinib, roscovitine, sorafenib and lapatinib as reference drugs (Table 2). Interestingly, the reported kinase inhibition results for compound **15** were compatible with that of the cytotoxicity assays (Table 1). This may explain the possible mechanism of cytotoxic action for the designed isatin–purine hybrid compounds. Furthermore, compound **15** exhibited inhibitory activities in the nanomolar ranges comparable to the positive controls against the four investigated protein kinases, with Her2 being the most sensitive, followed by EGFR, VEGFR2 and CDK2. The IC_50_ values of the kinase inhibitory activities were 0.15, 0.143, 0.534 and 0.192 μM, respectively.

#### 2.2.4. Molecular Docking

**Figure 3:**
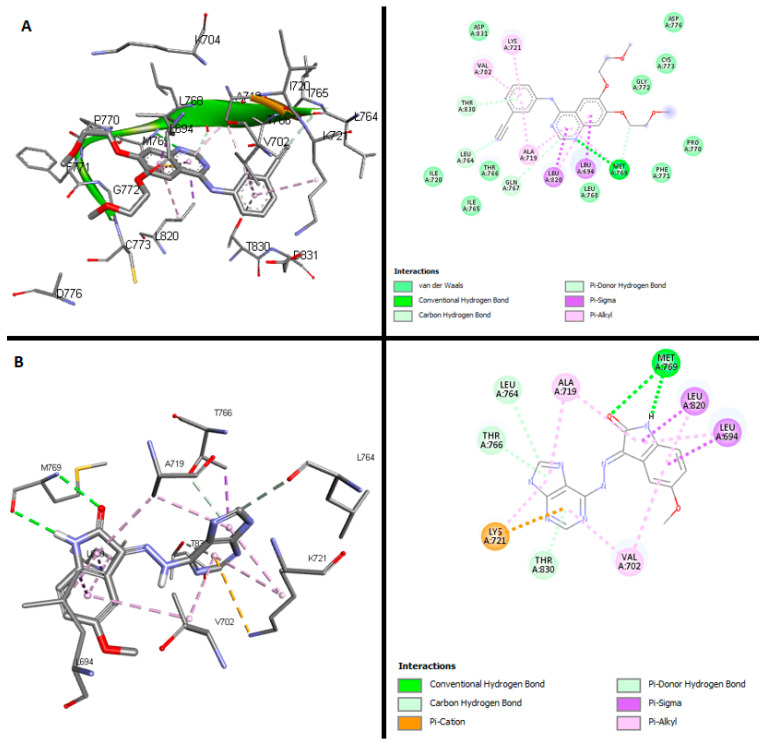
Schematic 2D and 3D representation of docking analysis of erlotinib (**A**) and compound **15** (**B**) in the active site of EGFR (PDB ID: 4HJO).

**Figure 4:**
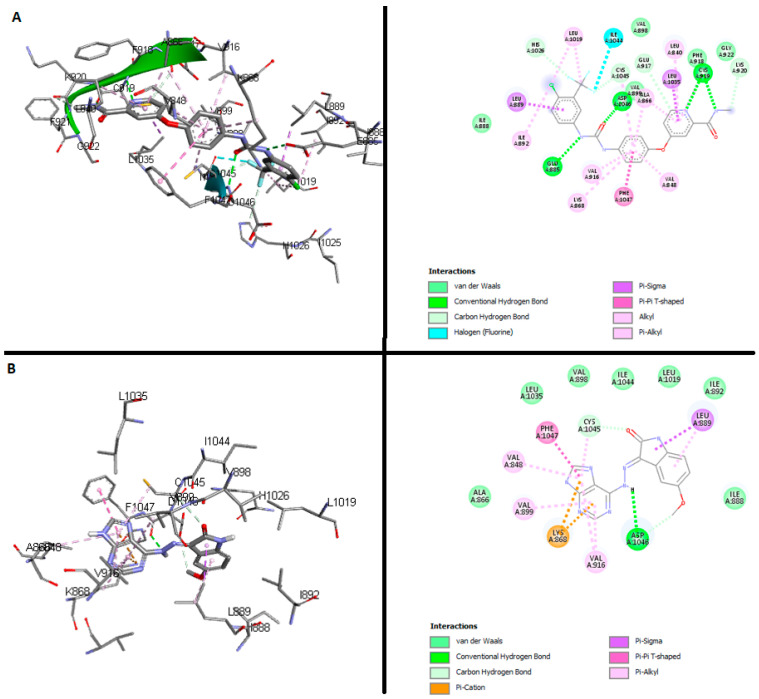
Schematic 2D and 3D representation of docking analysis of sorafenib (**A**) and compound **15** (**B**) in the active site of VEGFR2 (PDB ID: 4ASD).

**Figure 5:**
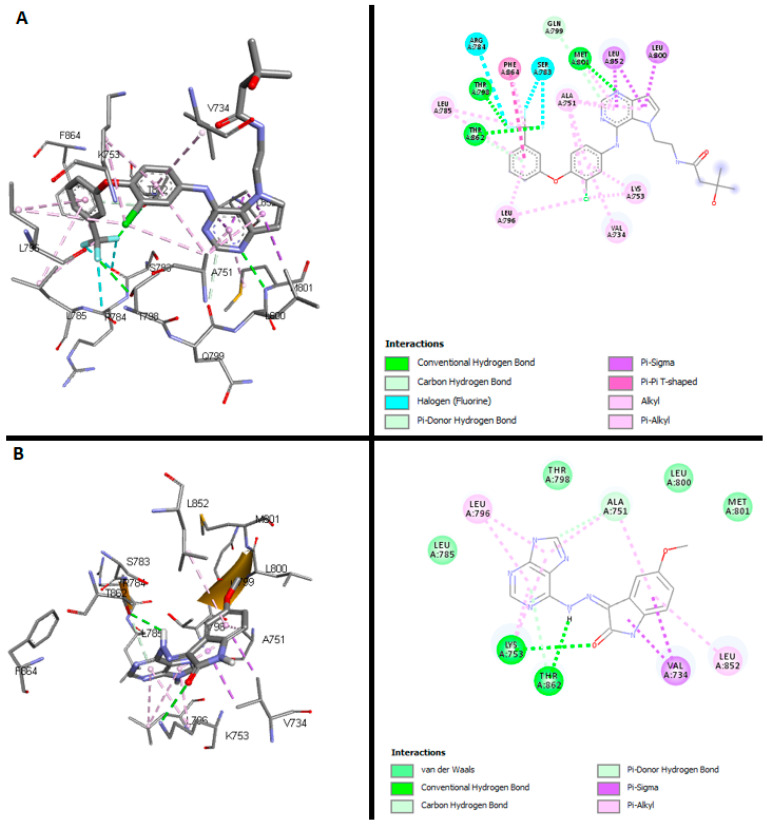
Schematic 2D and 3D representation of docking analysis of lapatinib (**A**) and compound **15** (**B**) in the active site of Her2 (PDB ID: 3RCD).

Compound **15** was docked in the active sites of EGFR, VEGFR2 and Her2 to rationalize its biological activity and to predict the possible types of drug–receptor interactions. Erlotinib, sorafenib and lapatinib were used as reference compounds, as they are the co-crystallized ligands in EGFR, VEGFR2 and Her2, respectively. Erlotinib was stabilized by a hydrogen bond with Met769 in the active site of EGFR. In addition, erlotinib had several hydrophobic interactions with Lys721, Val702, Ala719, Leu820 and Leu694, and two van der Waals interactions with Thr830 and Met769. On the other hand, compound **15** was stabilized by two hydrogen bonds between Met769 and the amide of the isatin moiety. Furthermore, compound **15** had multiple hydrophobic interactions with Ala719, Leu764, Thr766, Lys721, Thr830, Val702, Leu694 and Leu820 (Figure 3). The binding affinities for erlotinib and compound **15** with the EGFR were −9.5 and −8.9 Kcal/mol, respectively. Sorafenib, the reference compound for the docking experiment for VEGFR2, produced key interactions in the active site of VEGFR2 by forming hydrogen bonds with Cys919 (two hydrogen bonds), Asp1046 and Glu885, and several other types of interactions with the pocket’s amino acid residues. The hydrazine spacer of compound **15** formed a hydrogen bond with Asp1046, while fifteen hydrophobic interactions stabilized the compound in the active site of VEGFR2 (Figure 4). The binding affinities for sorafenib and compound **15** with VEGFR2 were −11.9 and −9.3 Kcal/mol, respectively. Regarding Her2, lapatinib formed three hydrogen bonds with amino acid residues, Met801, Thr798 and Thr862, and three halogen bonds with Ser783 (two) and Arg784 (one), and also had hydrophobic interactions with Leu800, Leu852, Met801, Ala751, Gln799, Ser783, Phe864, Arg784, Leu785, Leu796, Val 734 and Lys753. In contrast, Compound **15** bound the active site with two hydrogen bonds with Lys753 and The862, and had hydrophobic interactions with Ala751, Leu796, Lys753, Thr862, val734 and Leu 852 (Figure 5). The binding affinities for lapatinib and compound **15** with Her2 were −9.8 and −8.7 Kcal/mol, respectively.

#### 2.2.5. Cell Cycle Analysis and Apoptosis Rate

**Figure 6:**
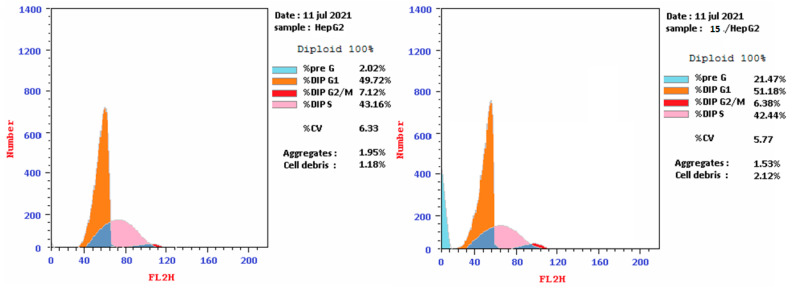
Flow cytometric analysis of cell cycle phases following treatment with compound **15**.

In addition, the cell cycle progression assay as described by Wang et al. [31,32] was used to investigate the effect of compound **15** in HepG2 cells. The biological effect of compound **15** was measured by treating HepG2 cells with 9.61 μM of the compound for 24 h. The compound’s effect on cell cycle distribution was analyzed by comparing with untreated control cells (Table 3, Figure 6). The results showed a massive accumulation of the treated HepG2 cells at the pre-G1 (21.47%, 10-fold) compared to the control cells (2.02%), revealed by flow cytometric analysis.

**Figure 7:**
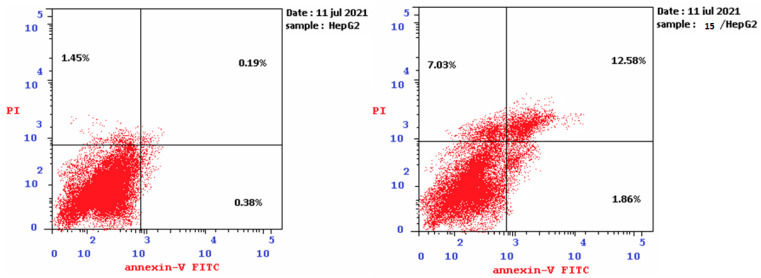
Flow cytometric analysis of apoptosis in HepG2 cells following treatment with compound **15**.

On the other hand, an Annexin V/propidium iodide (PI) double staining assay was conducted to identify the mode of cell death promoted by compound **15** (9.61 μM) following 24 h incubation with HepG2 cells. The results, in Table 4 and Figure 7, showed a notable increase in apoptotic cells in early-stage (0.38% to 1.86%) and late-stage (0.19% to 12.58%) apoptosis in the HepG2 cell line as compared to the controls. These results of the apoptosis induction effect, along with the previous results of cell cycle arrest, indicated the potential anti-cancer effect of compound **15**.

#### 2.2.6. BAX and Bcl-2, Caspase 3 and Caspase 9 Level Protein Assays 

We also investigated the effect of compound **15**, which was the most active compound, on protein expression levels of BAX, Bcl-2 Caspase 3 and Caspase 9. In these assays, compound **15** was used at its cytotoxic concentration (9.61 μM) and incubated with HepG2 cells for 24 h. The results showed an increase in the level of BAX, Caspase 3 and Caspase 9 proteins, with 3.36-, 3.90- and 3.11-fold increases, respectively, compared to the control cells (Table 5). In addition, Bcl-2 protein levels in the HepG2 cell significantly decreased, by 2.30-fold, after treatment with compound **15**, which affected the apoptosis pathway compared to the control.

## 3. Materials and Methods

### 3.1. Chemistry and Analysis

The ^1^H NMR spectrum was recorded with a Bruker 700 MHz NMR spectrometer in deuterated dimethyl sulfoxide (DMSO-d_6_) using tetramethylsilane (TMS) as the internal standard. Chemical shifts (δ) are reported in ppm, and J values are reported in Hz. The following abbreviations were used in reporting spectrum data: s (singlet), d (doublet), t (triplet), q (quartet), dd (doublet of doublets), td (triplet of doublets), and m (multiplet). An Agilent 6320 Ion Trap mass spectrometer (Agilent Technology, La Jolla, CA, USA) was used to obtain the mass spectra for all compounds.

#### 3.1.1. General Procedure A for Synthesis of Compounds **4**–**9**

A mixture of 4-((9*H*-purin-6-yl)amino)benzohydrazide (0.2 gm, 0.7 mmol), the appropriate isatin (0.7 mmol), and glacial acetic acid (1 mL) in absolute ethanol (20 mL) was refluxed for about 8 h. The reaction was then cooled and added to ice water. Afterwards, the resultant solid was filtered, rinsed with water and recrystallized from a proper organic solvent to achieve the desired compounds [19].

#### 3.1.2. General Procedure B for Synthesis of Compounds **11**–**16**

A mixture of 6-hydrazinyl-9H-purine 0.3 gm, 2 mmol), the appropriate isatin (0.7 mmol), and glacial acetic acid (1 mL) in absolute ethanol (20 mL) was refluxed for about 3–7 h. The reaction was then cooled and added to ice water. Afterwards, the resultant solid was filtered, rinsed with water and recrystallized from a proper organic solvent to obtain the desired compounds [19,33,34].

**Ethyl 4-((9H-purin-6-yl)amino)benzoate (2)**

Ethyl 4-aminobenzoate (26 mmol) was added to a solution of 6-chloro-9*H*-purine (13 mmol) in absolute ethanol (25 mL). The reaction mixture was stirred during reflux for 7 h. The solid obtained was filtered and washed with cold water to obtain the final product [34]. Yield 97.38%; m.p. > 300 °C; ^1^H NMR (700 MHz, DMSO-d_6_): δ 13.00 (s,1H), 10.21 (s, 1H), 8.49 (s, 1H), 8.37 (s, 1H), 8.19 (d, 2H), 7.94 (d, 2H), 4.78–3.90 (m, 2H), 1.59–0.97 (m, 3H). LC-MS *m*/*z* 281.9 (M^−^) 284.1 (M^+^); Anal. Calcd. for C_14_H_13_N_5_O_2_ (283.10): C, 59.36; H, 4.63; N, 24.72; Found C, 59.05; H, 4.56; N, 24.91%.

**4-((9H-purin-6-yl)amino)benzohydrazide (3)**

Ethyl 4-((*9H*-purin-6-yl)amino)benzoate (5 mmol) was refluxed in an excess of hydrazine hydrate for 3 h. The mixture was cooled and poured into ice-cold water. The resultant precipitate was filtered, washed thoroughly with water and dried to o the product [33]. Yield 84.14%; m.p. > 300 °C; ^1^H NMR (700 MHz, DMSO): δ 13.19 (s, 1H), 10.03 (s, 1H), 9.63 (s, 1H), 8.45 (s, 1H), 8.33 (s, 1H), 8.08–8.07 (d, 2H), 7.82–7.80 (d, 2H), 4.44 (s, 2H). LC-MS *m*/*z* 267.9 (M^−^) 270.0 (M^+^); Anal. Calcd. for C_14_H_13_N_5_O_2_ (269.10): C, 53.53; H, 4.12; N, 36.41; Found C, 53.17; H, 4.35; N 36.16, %.

**4-((9H-purin-6-yl)amino)-N’-(2-oxoindolin-3-ylidene)benzohydrazide (4)**

Synthesized according to general procedure A. Yield 90.20%; m.p. > 300 °C; ^1^H NMR (700 MHz, DMSO-d_6_): δ 13.97 (s, 1H), 13.29 (m, 1H), 11.48 (m, 1H), 10.28 (m, 1H), 8.61–8.16 (m, 5H), 7.89 (m, 1H), 7.63 (m, 1H), 7.41 (m, 1H), 7.13 (m, 1H), 6.96 (m, 1H). LC-MS *m*/*z* 396.9 (M^−^); Anal. Calcd. for C_20_H_14_N_8_O_2_ (398.12): C, 60.30; H, 3.54; N, 28.13; Found C, 59.88; H, 3.42; N, 27.85%.

**4-((9H-purin-6-yl)amino)-N’-(5-chloro-2-oxoindolin-3-ylidene)benzohydrazide (5)**

Synthesized according to general procedure A. Yield 70.72%; m.p. > 300 °C; 1H NMR (700 MHz, DMSO-d6): δ 13.51 (m, 1H), 11.52 (s, 1H), 10.29 (d, 2H), 8.76–6.59 (m, 9H). LC-MS *m*/*z* 430.9 (M^−^); C_20_H_13_ClN_8_O_2_ (432.08): C, 55.50; H, 3.03; N, 25.89; Found C, 55.75; H, 3.18; N, 25.61%.

**4-((9H-purin-6-yl)amino)-N’-(5-fluoro-2-oxoindolin-3-ylidene)benzohydrazide (6)**

Synthesized according to general procedure A. Yield 97.08%; m.p. > 300 °C; ^1^H NMR (700 MHz, DMSO-d6): δ 13.98 (s, 1H), 13.30 (d, 1H), 11.42 (s, 1H), 10.29 (d, 1H), 8.83–8.20 (m, 3H), 7.95 (m, 3H), 7.46 (s, 1H), 7.26 (s, 1H), 6.96 (m, 1H). LC-MS *m*/*z* 414.9 (M^−^); Anal. Calcd. for C_20_H_13_FN_8_O_2_ (416.11): C, 57.69; H, 3.15; N, 26.91; Found C,57.42; H, 3.13; N, 26.81%.

**4-((9H-purin-6-yl)amino)-N’-(5-methyl-2-oxoindolin-3-ylidene)benzohydrazide (7)**

Synthesized according to general procedure A. Yield 75.83%; m.p. > 300 °C; ^1^H NMR (700 MHz, DMSO-d_6_): δ 13.88 (m, 1H), 13.23 (m, 1H), 11.40 (m, 1H), 10.72 (s, 1H), 10.20 (m, 1H), 8.47 (m, 1H), 8.42–8.17 (m, 2H), 8.13–7.66 (m, 2H), 7.47 (s, 1H), 7.38–7.04 (m, 1H), 6.85 (m, 1H), 2.35 (s, 3H). LC-MS *m*/*z* 411.0 (M^−^); Anal. Calcd. for C_21_H_16_N_8_O_2_ (412.14): C, 61.16; H, 3.91; N, 27.17; Found C, 60.82; H, 3.83; N, 26.98%.

**4-((9H-purin-6-yl)amino)-N’-(5-methoxy-2-oxoindolin-3-ylidene)benzohydrazide (8)**

Synthesized according to general procedure A. Yield 96.82%; m.p. > 300 °C; ^1^H NMR (700 MHz, DMSO-d_6_): δ 14.04 (s, 1H), 13.30 (d, 1H), 11.22 (s, 1H), 10.48–10.09 (m, 1H), 8.83–7.61 (m, 6H), 7.17 (d, *J* = 2.1 Hz, 1H), 6.98 (d, 1H), 6.90 (d, *J* = 8.5 Hz, 1H), 3.77 (s, 3H, OCH_3_). LC-MS *m*/*z* 426.9 (M^−^); Anal. Calcd. For C_21_H_16_N_8_O_3_ (428.13): C, 58.88; H, 3.76; N, 26.16; Found C, 59.13; H, 3.90; N, 26.33%.

**4-((9H-purin-6-yl)amino)-N’-(5-nitro-2-oxoindolin-3-ylidene)benzohydrazide (9)**

Synthesized according to general procedure A. Yield 79.83%; m.p. > 300 °C; ^1^H NMR (700 MHz, DMSO-d_6_): δ 13.77 (s, 1H), 13.30 (m, 2H), 12.00 (d, 2H), 10.05 (s, 1H), 8.50–7.98 (m, 7H). LC-MS *m*/*z* 441.9 (M^−^); Anal. Calcd. for C_20_H_13_N_9_O_4_ (443.11): C, 54.18; H, 2.96; N, 28.43; Found C, 54.44; H, 3.13; N, 28.22%.

**6-hydrazineyl-9H-purine (10)**

6-chloro-9*H*-purine (10 mmol) was refluxed in an excess of hydrazine hydrate for 1 h. The mixture was cooled and poured into ice-cold water. The resultant precipitate was filtered, washed thoroughly with water and dried to obtain the product [33]. Yield 82.23%; m.p. 261–262 °C; ^1^H NMR (700 MHz, DMSO-d_6_): δ 12.94 (s, 1H), 12.22 (s, 1H), 9.15–8.40 (m, 1H), 8.40–7.81 (m, 1H), 4.32 (m, 1H), 1.37–1.03 (m, 1H).LC-MS *m*/*z* 149.0 (M^−^); Anal. Calcd. for C_5_H_6_N_6_ (150.1): C, 40.00; H, 4.03; N, 55.97; Found C, 39.55; H, 3.91; N, 55.66%.

**(E)-3-(2-(9H-purin-6-yl)hydrazineylidene)indolin-2-one (11)**

Synthesized according to general procedure B. Yield 88.33%; m.p. > 300 °C; ^1^H NMR (700 MHz, DMSO-d_6_): δ 12.54 (s, 1H), 11.29 (s, 1H), 10.53 (s, 1H), 8.67 (s, 1H), 8.31 (m, 1H), 8.10 (s, 1H), 7.34 (m, 1H), 7.20–6.76 (m, 2H).LC-MS *m*/*z* 277.9 (M^−^); Anal. Calcd. for C_13_H_9_N_7_O (279.1): C, 55.91; H, 3.25; N, 35.11; Found C, 56.17; H, 3.29; N, 35.34%.

**(E)-3-(2-(9H-purin-6-yl)hydrazineylidene)-5-chloroindolin-2-one (12)**

Synthesized according to general procedure B. Yield 99.5%; m.p. 296–298 °C; ^1^H NMR (700 MHz, DMSO-d_6_): δ 12.67 (s, 1H), 11.40 (s, 1H), 10.65 (s, 1H), 8.87–7.91 (m, 3H), 7.56–7.17 (m, 1H), 6.93 (m, 1H). LC-MS *m*/*z* 311.8 (M^−^); Anal. Calcd. for C_13_H_8_ClN_7_O (313.04): C, 49.77; H, 2.57; N, 31.26; Found C, 49.49; H, 2.40; N, 31.39%.

**(E)-3-(2-(9H-purin-6-yl)hydrazineylidene)-5-fluoroindolin-2-one (13)**

Synthesized according to general procedure B. Yield 97.94%; m.p. > 300 °C; ^1^H NMR (700 MHz, DMSO-d6): δ 13.22 (s, 1H), 12.59 (s, 1H), 11.31 (s, 1H), 8.57 (d, 1H), 8.19 (m, 2H), 7.61–7.09 (m, 1H), 6.91 (m, 1H). LC-MS *m*/*z* 295.9 (M^−^) 298.1 (M^+^); Anal. Calcd. for C_13_H_8_FN_7_O (297.1): C, 52.53; H, 2.71; N, 32.98; Found C, 52.90; H, 2.58; N, 32.57%.

**(E)-3-(2-(9H-purin-6-yl)hydrazineylidene)-5-methylindolin-2-one (14)**

Synthesized according to general procedure B. Yield 93.69%; m.p. 294–296 °C; ^1^H NMR (700 MHz, DMSO-d_6_): δ 12.39 (m, 1H), 11.18 (m, 1H), 10.49 (m, 1H), 9.12–7.88 (m, 3H), 7.34–6.98 (m, 1H), 6.81 (m, 1H), 2.42–2.23 (m, 3H). LC-MS *m*/*z* 291.9 (M^−^) 294.1 (M^+^); Anal. Anal. Calcd. for C_14_H_11_N_7_O (293.1): C, 57.33; H, 3.78; N, 33.43; Found C, 57.61; H, 4.05; N, 33.29%.

**(E)-3-(2-(9H-purin-6-yl)hydrazineylidene)-5-methoxyindolin-2-one (15)**

Synthesized according to general procedure B. Yield 95.19%; m.p. > 300 °C; ^1^H NMR (700 MHz, DMSO-d_6_): δ 12.53 (s, 1H), 11.43–10.82 (m, 1H), 10.43 (m, 1H), 8.54 (m, 1H), 8.17 (m, 1H), 7.30–6.32 (m, 3H), 3.79 (s, 3H). LC-MS *m*/*z* 307.9 (M^−^) 310.1 (M^+^); Anal. Calcd. for C_14_H_11_N_7_O_2_ (309.1): C, 54.37; H, 3.58; N, 31.70; Found C, 54.61; H, 3.64; N, 31.58%.

**(E)-3-(2-(9H-purin-6-yl)hydrazineylidene)-5-nitroindolin-2-one (16)**

Synthesized according to general procedure B. Yield 72.46%; m.p. > 300 °C; ^1^H NMR (700 MHz, DMSO-d_6_): δ 12.67 (s, 1H), 11.40 (s, 1H), 10.65 (s, 1H), 8.87–7.91 (m, 3H), 7.56–7.17 (m, 1H), 6.93 (m, 1H). LC-MS *m*/*z* 322.9 (M^−^); Anal. Calcd. for C_13_H_8_N_8_O_3_ (324.1): C, 48.15; H, 2.49; N, 34.56; Found C, 47.82; H, 2.23; N, 34.30%.

### 3.2. Biological Evaluation

#### 3.2.1. Cytotoxicity Assay

Following the reported method [35], the cytotoxicity of the synthesized 12 compounds, in addition to Sunitinib and Doxorubicin, was evaluated using an MTT assay against HePG-2, MCF-7, MDA-MB-231 and HeLa cancer cell lines. The cells were seeded in an RPMI-11640 medium with 10% fetal bovine serum (FBS) and an antibiotic cocktail of 100 μL/mL streptomycin and 100 units/mL penicillin. Cancer cell lines were cultured separately in 96-well plates at a concentration of 1.0 × 10^4^ cells/well at 37 °C, 5% CO_2_, and 100% relative humidity. After incubation for 48 h, the cells were treated with different concentrations of the synthesized hybrid compounds for 24 h. After the addition of 20 μL MTT (5 mg/mL), the plates were incubated for 4 h and then 100 μL Dimethyl sulfoxide (DMSO) was added to each well to dissolve the insoluble purple formazan formed. Finally, the absorbance was measured at 570 nm using a BioTek EXL 800 plate reader (Agilent Technologies, Inc., Santa Clara, CA, USA). The relative cell viability percentage was calculated as (A570 of treated samples/A570 of untreated sample) × 100.

#### 3.2.2. In Vitro CDK2, EGFR, Her2, and VEGFR-2 Enzyme Assays

Compound **15** was evaluated for inhibitory activity against EGFR, VEGFR-2, Her2 and CDK2. A human ELISA kit (Enzyme-Linked Immunosorbent Assay) for each of the above-mentioned protein kinase enzymes was used in this assay. First, a specific antibody for each enzyme was added separately to a 96-well plate, and 100 μL of the standard solution or the compound **15** was added. The mixture was then incubated at room temperature for 2.5 h, after which the wells were washed and 100 μL of the prepared biotin antibody was added and incubated for 1 h at room temperature. After another washing step, 100 μL of streptavidin solution was added and incubated for 45 min at room temperature. The wells were then washed for a third time and 100 μL of TMB Substrate reagent was added and incubated for 30 min at room temperature. Finally, 50 μL of the stop solution was added, and the absorbance was read immediately at 450 nm. A standard curve was plotted showing concentration on the *X*-axis and absorbance on the *Y*-axis [14,36,37,38,39].

#### 3.2.3. Flow Cytometry Analysis for Cell Cycle

Flow cytometry analysis was carried out using an ab139418 Propidium Iodide flow cytometry kit/BD to detect the effect of the synthesized compound **15** on cell cycle distribution. First, HepG2 cell were seeded in 6-well plates at a density of 2 × 10^5^/well and incubated for 24 h. HepG2 cells were then treated with 9.61 µM of compound **15** for 24 h. The treated cells were then fixed in 70% ethanol for 12 h at 4 °C, rinsed with cold PBS, incubated with 100 μL RNase A for 0.5 h at 37 °C, and stained with Propidium Iodide (400 μL) in the dark at RT for an extra 0.5 h. The stained cells were determined utilizing Epics XLMCL™ flow cytometer equipment (Beckman Coulter, Apeldoorn, Netherlands), and the results were collected and analyzed using Flowing software (version 2.5.1, Turku Centre for Biotechnology, Turku, Finland).

#### 3.2.4. Flow Cytometry Analysis for Apoptosis

Flow cytometry cell apoptosis analysis was conducted to examine the apoptotic effect of the synthesized compounds using the Annexin V-FITC cell apoptosis detection kit (Biovision, K101-100). First, HepG2 cells in a density of 2 × 10^5^ were seeded in 6-well plates and incubated for 24 h. The cells were then treated with 9.61 µM compound **15** for 24 h. The treated cells were then detached with trypsin (5 min, 37 °C), collected by centrifugation (5 min, 300× *g*), washed twice with PBS, and resuspended in 0.1 mL of a 1X binding buffer. After that, the cells were double-stained with 5 μL Annexin V-FITC and 5 μL PI in the dark at room temperature for 15 min. The stained cells were evaluated using an Epics XL-MCL™ Flow Cytometer (Beckman Coulter, Apeldoorn, Netherlands) with an excitation wavelength of 488 nm and an emission wavelength of 530 nm, and the data were interpreted using the Flowing software (version 2.5.1, Turku Centre for Biotechnology, Turku, Finland).

#### 3.2.5. Determination of BAX, Bcl-2, Caspase 3 and Caspase 9 Levels

HepG2 cells were seeded in 96-well plates in triplicate and incubated for 24 h. The incubated cells were then treated with compound **15** at different concentrations. Cells treated with 0.1% DMSO (V/V) served as control. After 24 h incubation, caspase 3 and 9, BAX and Bcl-2 levels were determined using ELISA assay kits KHO1091 (Invitrogen^TM^, Grand Island, NY, USA), EIA-4860 (DRU International INC., Mountainside, NJ, USA), EIA-4487 (DRU International INC., Mountainside, NJ, USA) and 99-0042 (Invitrogen^TM^, Grand Island, NY, USA), respectively, according to the manufacturers’ procedures.

#### 3.2.6. Molecular Docking

The PDB Data Bank (http://www.rcsb.org, (accessed on 25 December 2022) was utilized to obtain the X-ray crystal structures of the EGFR kinase domain in complex with erlotinib (PDB ID: 4HJO), the VEGFR2 kinase domain in complex with sorafenib (PDB ID:4ASD) and the kinase domain of human HER2 (PDB ID: 3RCD) in complex with lapatinib. The Discovery Studio, AutoDock Tools, Vina, and PyRx (The Scripps Research Institute, La Jolla, CA) software programs were used for docking simulations. First, the protein crystal structures were prepared by removing all additional molecules such as water, ligands, and sulfate. The generated data was saved in PDB file format. Second, AutoDock Tools was used to add polar hydrogens to the previous file saved in the PDB format, and the data were saved in PDBQT format. Third, using Discovery Studio, the co-crystallized ligands were separated from the protein structure and saved in a PDB file. Compound **15** was also saved in a PDB file. A grid box was employed to eliminate nonspecific binding interactions and minimize docking time. Finally, PyRx was used to perform the docking simulations, while the lowest energy pose of compound **15** superimposed with the co-crystallized ligands was used to study the interaction with the kinase enzyme.

## 4. Conclusions

Isatin–purine hybrid compounds were designed, synthesized and biologically evaluated successfully. Some of these compounds showed potency comparable to the reference compounds in inhibiting cell proliferation against HepG2, MCF-7, MDA-MB-231 and HeLa cell lines. In particular, compound **15**, with a methoxy substitution and a hydrazine linker, retained good activity in the nanomolar range in the inhibition of EGFR, Her2, VEGFR2 and CDK2 compared to the reference compounds. In addition, cell cycle analysis and BAX, Bcl-2, Caspase 3 and Caspase 9 protein level determination assays indicated the apoptosis-inducing effect of compound **15**. Overall, this work presents the isatin–purine hybrid compounds as novel compounds targeting multi-kinases that may prove useful in the discovery of new anticancer therapeutics.

## Figures and Tables

**Table 1:**
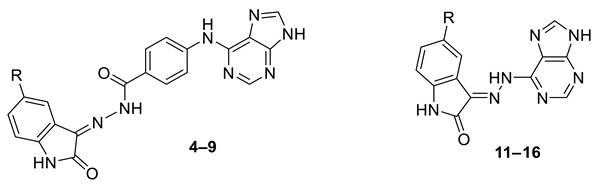
Cytotoxicity effect of isatin–purine hybrid compounds **4**–**16** on HepG2, MCF-7, MDA-MB-231 and HeLa.

Compound	R	In Vitro Cytotoxicity IC_50_ (µM)			
		HepG2	MCF-7	MDA-MB-231	HeLa
**4**	-	54.62 ± 3.1	47.26 ± 3.0	59.67 ± 2.9	62.38 ± 3.4
**5**	Cl	88.14 ± 4.0	>100	82.13 ± 3.8	>100
**6**	F	42.35 ± 2.7	22.31 ± 1.8	19.05 ± 1.4	30.69 ± 2.3
**7**	CH_3_	60.38 ± 3.2	76.38 ± 3.7	91.60 ± 4.5	>100
**8**	OCH_3_	48.12 ± 2.9	40.57 ± 2.7	29.78 ± 2.0	53.49 ± 3.2
**9**	NO_2_	75.56 ± 3.5	65.01 ± 3.4	72.24 ± 3.4	84.57 ± 4.1
**11**	-	12.89 ± 1.0	15.60 ± 1.3	24.83 ± 1.9	17.63 ± 1.4
**12**	Cl	26.45 ± 1.8	33.04 ± 2.3	41.29 ± 2.5	46.72 ± 2.8
**13**	F	31.70 ± 2.1	38.82 ± 2.5	45.21 ± 2.7	51.20 ± 3.0
**14**	CH_3_	18.06 ± 1.3	27.53 ± 2.1	32.55 ± 2.2	43.51 ± 2.7
**15**	OCH_3_	9.61 ± 0.8	10.78 ± 0.9	14.89 ± 1.2	8.93 ± 0.8
**16**	NO_2_	39.73 ± 2.5	58.32 ± 3.2	67.42 ± 3.2	69.50 ± 3.6
Sunitinib	-	6.82 ± 0.5	5.19 ± 0.4	8.41 ± 0.7	7.48 ± 0.6

All IC_50_ values are presented as the mean ± SD of three different experiments.

**Table 2:** Kinase inhibitory effects of compound **15** on EGFR, Her2, VEGFR2 and CDK2.

Compound	Kinase Protein	IC_50_ (μM)
**15**	CDK2	0.534
Roscovitine		0.143
**15**	EGFR	0.143
Erlotinib		0.041
**15**	Her2	0.15
Lapatinib		0.051
**15**	VEGFR2	0.192
Sorafenib		0.049

All IC_50_ values are presented as the mean ± SD of three different experiments.

**Table 3:** Effect of compound **15** on cell cycle progression in HepG2 cells.

Compound/Cell Line	Cell Cycle Distribution (%)			
	% G0–G1	% S	% G2/M	% Pre-G1
Compound **15**/HepG2	51.18	42.44	6.38	21.47
Cont. HepG2	44.39	38.16	17.45	2.02

All percentages are presented as the mean of three different experiments.

**Table 4:** Effect of compound **15** on the distribution of apoptotic cells in HepG2 cells.

Sample	Apoptosis			
	Viable	Early	Late	Necrosis
Compound **15**/HepG2	78.53%	1.86%	12.58%	7.03%
Cont. HepG2	97.98%	0.38%	0.19%	1.45%

All percentages are presented as the mean of three different experiments.

**Table 5:** Effect of compound **15** on protein expression of Bax, Bcl-2, Caspase 3 and Caspase 9.

Compound/Cell Line	Protein	Expression (Pg/mL)	Fold
**15**/HepG2	BAX	279.1 ± 8.15	3.38
Control HepG2		83.88 ± 23	1
**15**/HepG2	Bcl-2	4.593 ± 0.22	0.43
Control HepG2		10.67 ± 0.83	1
**15**/HepG2	Caspase 3	368.4 ± 20.7	3.95
Control HepG2		93.23 ± 15.5	1
**15**/HepG2	Caspase 9	14.07 ± 0.58	3.11
Control HepG2		4.52 ± 1.27	1

All percentages are presented as the mean ± SD of three different experiments.

## Data Availability

Not applicable.

## Supplementary Materials

The following are available online at https://www.mdpi.com/article/10.3390/medicina59030610/s1; ^1^HNMR and Mass spectra for compound **4**–**16**.

## References

[1] Alanazi M.M., Mahdy H.A., Alsaif N.A., Obaidullah A.J., Alkahtani H.M., Al-Mehizia A.A., Alsubaie S.M., Dahab M.A., Eissa I.H. (2021). New Bis([1,2,4]Triazolo)[4,3-a:3′,4′-c]Quinoxaline Derivatives as VEGFR-2 Inhibitors and Apoptosis Inducers: Design, Synthesis, in Silico Studies, and Anticancer Evaluation. Bioorg. Chem..

[2] Alanazi M.M., Aldawas S., Alsaif N.A. (2023). Design, Synthesis, and Biological Evaluation of 2-Mercaptobenzoxazole Derivatives as Potential Multi-Kinase Inhibitors. Pharmaceuticals.

[3] Ferlay J., Colombet M., Soerjomataram I., Parkin D.M., Piñeros M., Znaor A., Bray F. (2021). Cancer Statistics for the Year 2020: An Overview. Int. J. Cancer.

[4] AACR Cancer Progress Report—Cancer Progress Report. https://cancerprogressreport.aacr.org/progress/.

[5] El-Azab A.S., Abdel-Aziz A.A.M., AlSaif N.A., Alkahtani H.M., Alanazi M.M., Obaidullah A.J., Eskandrani R.O., Alharbi A. (2020). Antitumor Activity, Multitarget Mechanisms and Molecular Docking Studies of Quinazoline Derivatives Based on a Benzenesulfonamide Scaffold: Cell Cycle Analysis. Bioorg. Chem..

[6] Alsaif N.A., Dahab M.A., Alanazi M.M., Obaidullah A.J., Al-Mehizia A.A., Alanazi M.M., Aldawas S., Mahdy H.A., Elkady H. (2021). New Quinoxaline Derivatives as VEGFR-2 Inhibitors with Anticancer and Apoptotic Activity: Design, Molecular Modeling, and Synthesis. Bioorg. Chem..

[7] Szakács G., Paterson J.K., Ludwig J.A., Booth-Genthe C., Gottesman M.M. (2006). Targeting Multidrug Resistance in Cancer. Nat. Rev. Drug Discov..

[8] Fu R.G., Sun Y., Sheng W.B., Liao D.F. (2017). Designing Multi-Targeted Agents: An Emerging Anticancer Drug Discovery Paradigm. Eur. J. Med. Chem..

[9] Abdel-Aziz A.A.-M., El-Azab A.S., AlSaif N.A., Obaidullah A.J., Al-Obaid A.M., Al-Suwaidan I.A. (2021). Synthesis, Potential Antitumor Activity, Cell Cycle Analysis, and Multitarget Mechanisms of Novel Hydrazones Incorporating a 4-Methylsulfonylbenzene Scaffold: A Molecular Docking Study. J. Enzyme Inhib. Med. Chem..

[10] Schlesinger J., Ullricht A. (1992). Growth Factor Signaling by Receptor Tyrosine Kinases Review. Neuron.

[11] Hanks S.K., Quinn A.M., Hunter T. (1988). The Protein Kinase Family: Conserved Features and Deduced Phylogeny of the Catalytic Domains. Science.

[12] El-Husseiny W.M., El-Sayed M.A.A., El-Azab A.S., AlSaif N.A., Alanazi M.M., Abdel-Aziz A.A.M. (2020). Synthesis, Antitumor Activity, and Molecular Docking Study of 2-Cyclopentyloxyanisole Derivatives: Mechanistic Study of Enzyme Inhibition. J. Enzyme Inhib. Med. Chem..

[13] Alkahtani H.M., Abdalla A.N., Obaidullah A.J., Alanazi M.M., Almehizia A.A., Alanazi M.G., Ahmed A.Y., Alwassil O.I., Darwish H.W., Abdel-Aziz A.A.M. (2020). Synthesis, Cytotoxic Evaluation, and Molecular Docking Studies of Novel Quinazoline Derivatives with Benzenesulfonamide and Anilide Tails: Dual Inhibitors of EGFR/HER2. Bioorg. Chem..

[14] Fontanella C., Ongaro E., Bolzonello S., Guardascione M., Fasola G., Aprile G. (2014). Clinical Advances in the Development of Novel VEGFR2 Inhibitors. Ann. Transl. Med..

[15] Bray F., Ferlay J., Soerjomataram I., Siegel R.L., Torre L.A., Jemal A. (2018). Global Cancer Statistics 2018: GLOBOCAN Estimates of Incidence and Mortality Worldwide for 36 Cancers in 185 Countries. CA Cancer J. Clin..

[16] Alanazi M.M., Elkady H., Alsaif N.A., Obaidullah A.J., Alkahtani H.M., Alanazi M.M., Alharbi M.A., Eissa I.H., Dahab M.A. (2021). New Quinoxaline-Based VEGFR-2 Inhibitors: Design, Synthesis, and Antiproliferative Evaluation with in Silico Docking, ADMET, Toxicity, and DFT Studies. RSC Adv..

[17] Alanazi M.M., Eissa I.H., Alsaif N.A., Obaidullah A.J., Alanazi W.A., Alasmari A.F., Albassam H., Elkady H., Elwan A. (2021). Design, Synthesis, Docking, ADMET Studies, and Anticancer Evaluation of New 3-Methylquinoxaline Derivatives as VEGFR-2 Inhibitors and Apoptosis Inducers. J. Enzyme Inhib. Med. Chem..

[18] Alanazi M.M., Alaa E., Alsaif N.A., Obaidullah A.J., Alkahtani H.M., Al-Mehizia A.A., Alsubaie S.M., Taghour M.S., Eissa I.H. (2021). Discovery of New 3-Methylquinoxalines as Potential Anti-Cancer Agents and Apoptosis Inducers Targeting VEGFR-2: Design, Synthesis, and in Silico Studies. J. Enzyme Inhib. Med. Chem..

[19] Alkahtani H.M., Alanazi M.M., Aleanizy F.S., Alqahtani F.Y., Alhoshani A., Alanazi F.E., Almehizia A.A., Abdalla A.N., Alanazi M.G., El-Azab A.S. (2019). Synthesis, Anticancer, Apoptosis-Inducing Activities and EGFR and VEGFR2 Assay Mechanistic Studies of 5,5-Diphenylimidazolidine-2,4-Dione Derivatives: Molecular Docking Studies. Saudi Pharm. J..

[20] Al-Sanea M.M., Obaidullah A.J., Shaker M.E., Chilingaryan G., Alanazi M.M., Alsaif N.A., Alkahtani H.M., Alsubaie S.A., Abdelgawad M.A. (2021). A New CDK2 Inhibitor with 3-Hydrazonoindolin-2-One Scaffold Endowed with Anti-Breast Cancer Activity: Design, Synthesis, Biological Evaluation, and In Silico Insights. Molecules.

[21] Mohamed A.R., El Kerdawy A.M., George R.F., Georgey H.H., Abdel Gawad N.M. (2021). Design, Synthesis and in Silico Insights of New 7,8-Disubstituted-1,3-Dimethyl-1H-Purine-2,6(3H,7H)-Dione Derivatives with Potent Anticancer and Multi-Kinase Inhibitory Activities. Bioorg. Chem..

[22] Wang X., Han C., Wu K., Luo L., Wang Y., Du X., He Q., Ye F. (2018). Design, Synthesis and Ability of Non-Gold Complexed Substituted Purine Derivatives to Inhibit LPS-Induced Inflammatory Response. Eur. J. Med. Chem..

[23] Oyarzabal J., Zarich N., Albarran M.I., Palacios I., Urbano-Cuadrado M., Mateos G., Reymundo I., Rabal O., Salgado A., Corrionero A. (2010). Discovery of Mitogen-Activated Protein Kinase-Interacting Kinase 1 Inhibitors by a Comprehensive Fragment-Oriented Virtual Screening Approach. J. Med. Chem..

[24] Nepali K., Chang T.Y., Lai M.J., Hsu K.C., Yen Y., Lin T.E., Lee S.B., Liou J.P. (2020). Purine/Purine Isoster Based Scaffolds as New Derivatives of Benzamide Class of HDAC Inhibitors. Eur. J. Med. Chem..

[25] Hu J., Han Y., Wang J., Liu Y., Zhao Y., Liu Y., Gong P. (2018). Discovery of Selective EGFR Modulator to Inhibit L858R/T790M Double Mutants Bearing a N-9-Diphenyl-9H-Purin-2-Amine Scaffold. Bioorg. Med. Chem..

[26] Laufer S.A., Domeyer D.M., Scior T.R.F., Albrecht W., Hauser D.R.J. (2005). Synthesis and Biological Testing of Purine Derivatives as Potential ATP-Competitive Kinase Inhibitors. J. Med. Chem..

[27] Anscombe E., Meschini E., Mora-Vidal R., Martin M.P., Staunton D., Geitmann M., Danielson U.H., Stanley W.A., Wang L.Z., Reuillon T. (2015). Identification and Characterization of an Irreversible Inhibitor of CDK2. Chem. Biol..

[28] Bhat M., Robichaud N., Hulea L., Sonenberg N., Pelletier J., Topisirovic I. (2015). Targeting the Translation Machinery in Cancer. Nat. Rev. Drug Discov..

[29] Lineham E., Spencer J., Morley S.J. (2017). Dual Abrogation of MNK and MTOR: A Novel Therapeutic Approach for the Treatment of Aggressive Cancers. Future Med. Chem..

[30] Ding Z., Zhou M., Zeng C. (2020). Recent Advances in Isatin Hybrids as Potential Anticancer Agents. Arch. Pharm..

[31] Wang J., Lenardo M.J. (2000). Roles of Caspases in Apoptosis, Development, and Cytokine Maturation Revealed by Homozygous Gene Deficiencies. J. Cell Sci..

[32] Riccardi C., Nicoletti I. (2006). Analysis of Apoptosis by Propidium Iodide Staining and Flow Cytometry. Nat. Protoc..

[33] Mohameda M.S., Kamel M.M., Kassem E.M., Abotaleb N., AbdEl-Moez S.I., Ahmed M.F. (2010). Novel 6,8-Dibromo-4(3H)Quinazolinone Derivatives of Anti-Bacterial and Anti-Fungal Activities. Eur. J. Med. Chem..

[34] Musumeci F., Fallacara A.L., Brullo C., Grossi G., Botta L., Calandro P., Chiariello M., Kissova M., Crespan E., Maga G. (2021). Identification of New Pyrrolo[2,3-d]Pyrimidines as Src Tyrosine Kinase Inhibitors in Vitro Active against Glioblastoma. Eur. J. Med. Chem..

[35] van Meerloo J., Kaspers G.J.L., Cloos J. (2011). Cell Sensitivity Assays: The MTT Assay. Methods Mol. Biol..

[36] Asghar U., Witkiewicz A.K., Turner N.C., Knudsen E.S. (2015). The History and Future of Targeting Cyclin-Dependent Kinases in Cancer Therapy. Nat. Rev. Drug Discov..

[37] Tai W., Mahato R., Cheng K. (2010). The Role of HER2 in Cancer Therapy and Targeted Drug Delivery. J. Control. Release.

[38] Nakamura J.L. (2007). The Epidermal Growth Factor Receptor in Malignant Gliomas: Pathogenesis and Therapeutic Implications. Expert Opin. Ther. Targ..

[39] Sharma K., Suresh P.S., Mullangi R., Srinivas N.R. (2015). Quantitation of VEGFR2 (Vascular Endothelial Growth Factor Receptor) Inhibitors--Review of Assay Methodologies and Perspectives. Biomed. Chromatogr..

