# Supplementary material for: Antiproliferative Activity, Multikinase Inhibition, Apoptosis- Inducing Effects and Molecular Docking of Novel Isatin–Purine Hybrids
Source: Medicina (Kaunas). 2023 Mar 19;59(3):610. doi: 10.3390/medicina59030610 (PMC10051310; doi:10.3390/medicina59030610)
Supplement: Supplementary file 1 [file medicina-59-00610-s001.zip › medicina-2239020-supplementary.pdf]

Supplementary data of

Anti-proliferative activity, multi-kinase inhibition, apoptosis- inducing effects and molecular docking of novel isatin-purine hybrids

Ashwag S. Alanazi[1]*, Tebyan O. Mirgany[2], Aisha A. Alsfouk[1], Nawaf A. Alsaif[2] and Mohammed M. Alanazi[2]*

[1] Department of Pharmaceutical Sciences, College of Pharmacy, Princess Nourah Bint Abdulrahman University, Riyadh 84428, Saudi Arabia

[2] Department of Pharmaceutical Chemistry, College of Pharmacy, King Saud University, P.O. Box 2457, Riyadh 11451, Saudi Arabia

<u>Compound 2:</u>

ethyl 4-((9H-purin-6-yl)amino)benzoate (2)

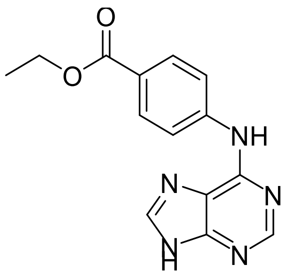

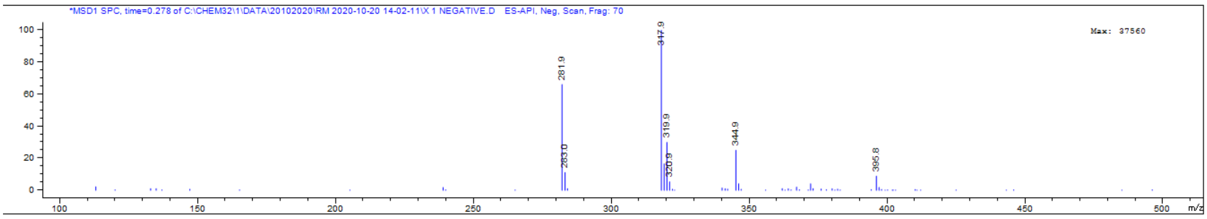

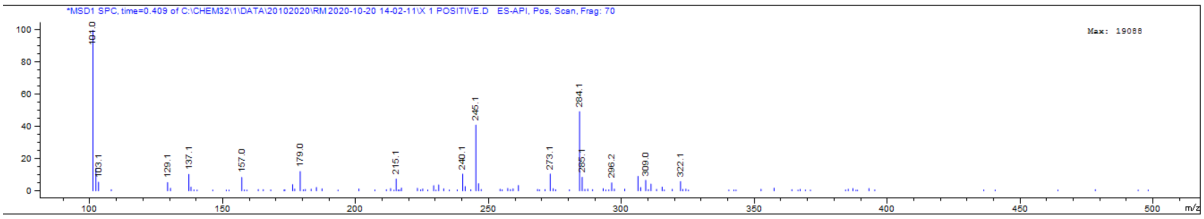

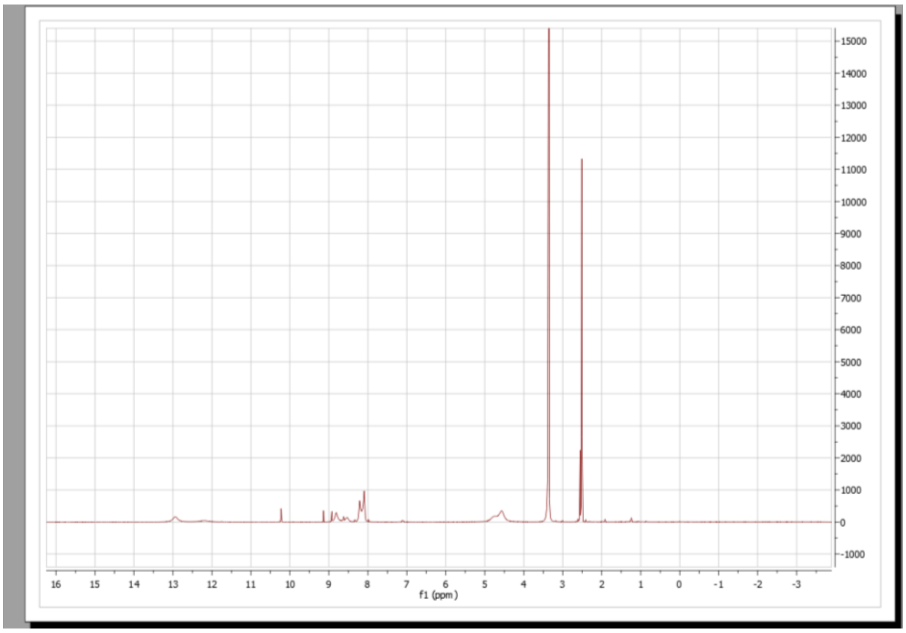

Compound 3:

4-((9H-purin-6-yl)amino)benzohydrazide (3)

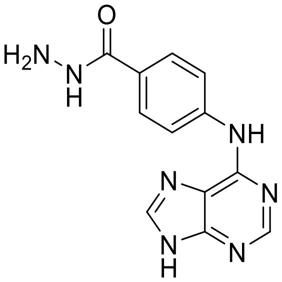

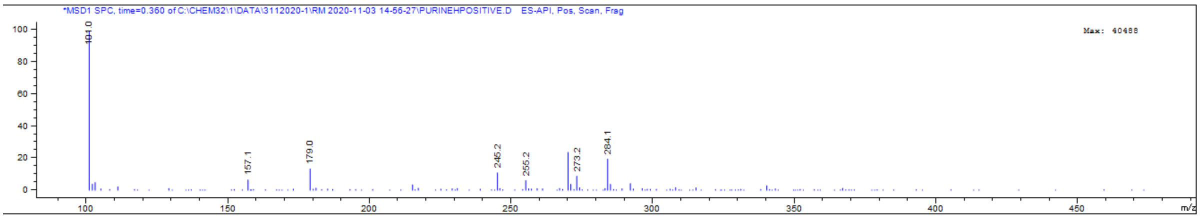

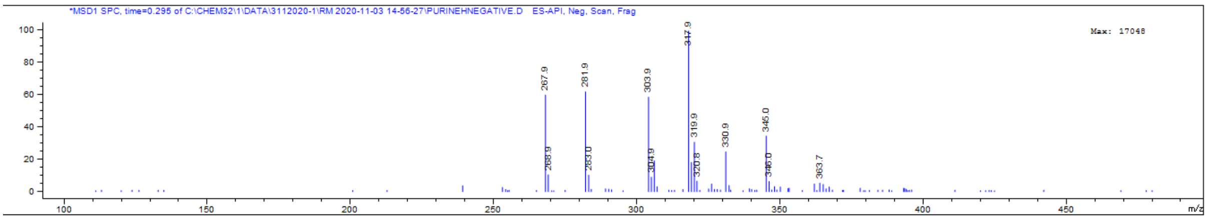

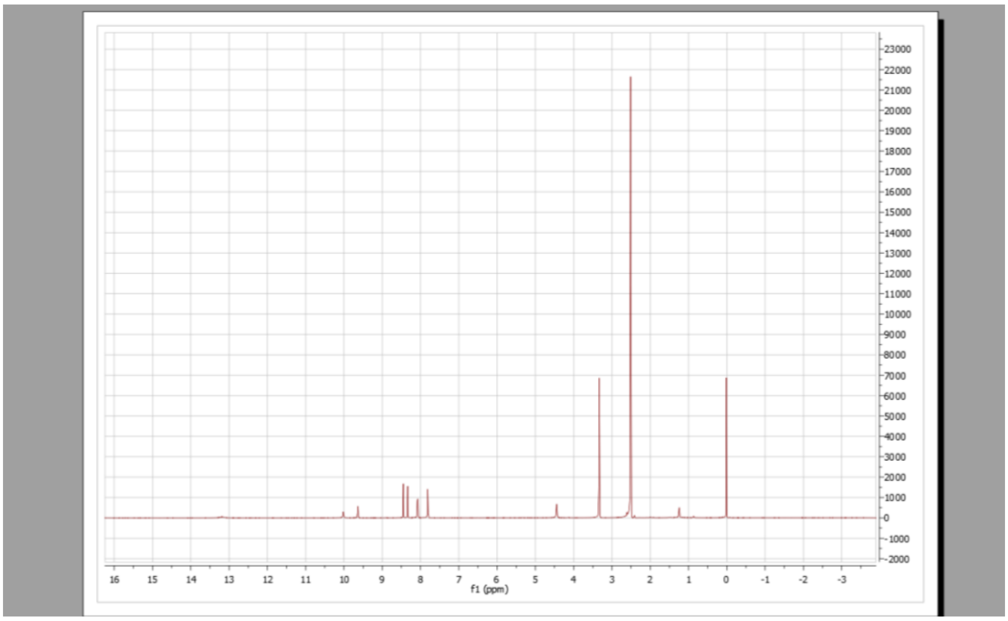

Compound 4:

4-((9H-purin-6-yl)amino)-N'-(2-oxoindolin-3-ylidene)benzohydrazide (4)

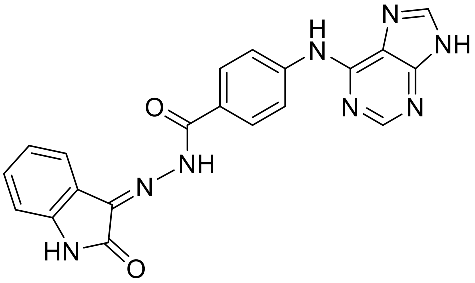

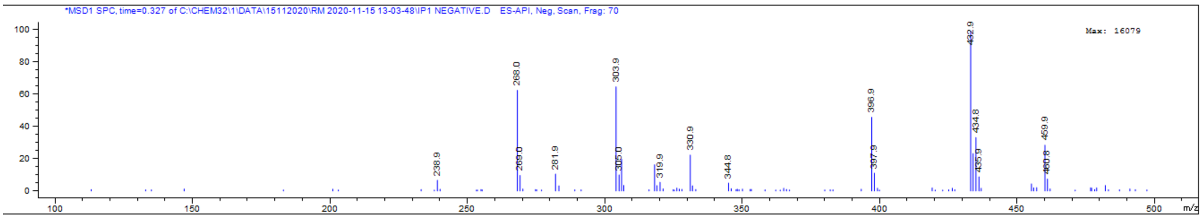

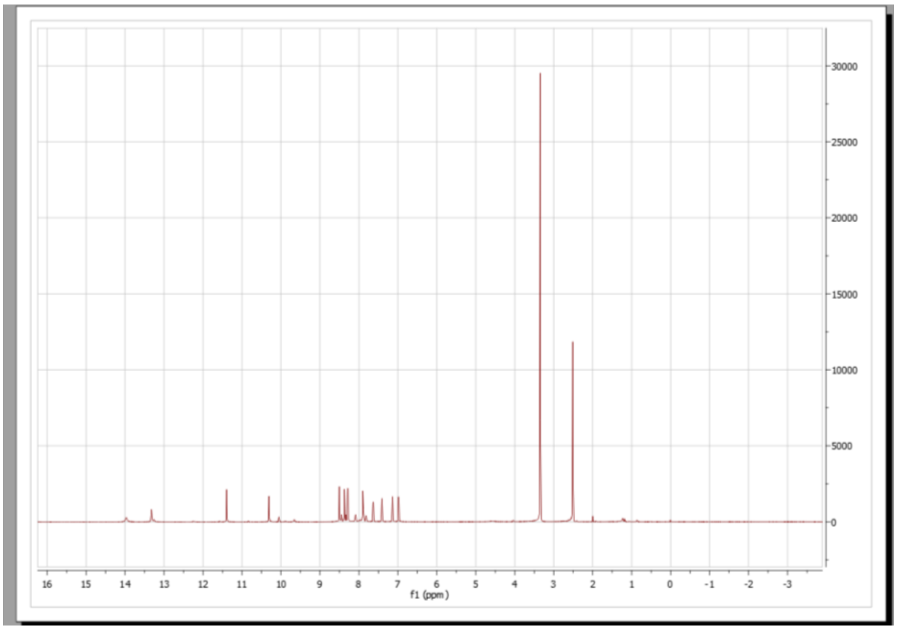

Compound 5:

4-((9H-purin-6-yl)amino)-N'-(5-chloro-2-oxoindolin-3-ylidene)benzohydrazide (5)

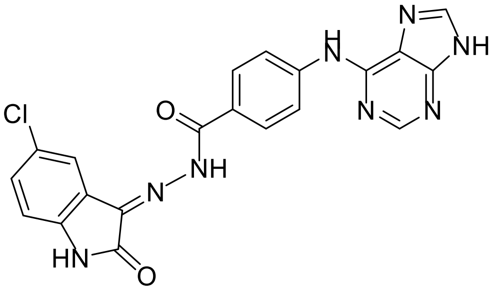

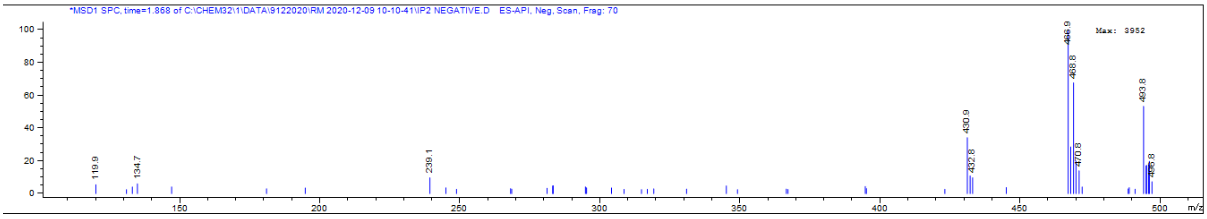

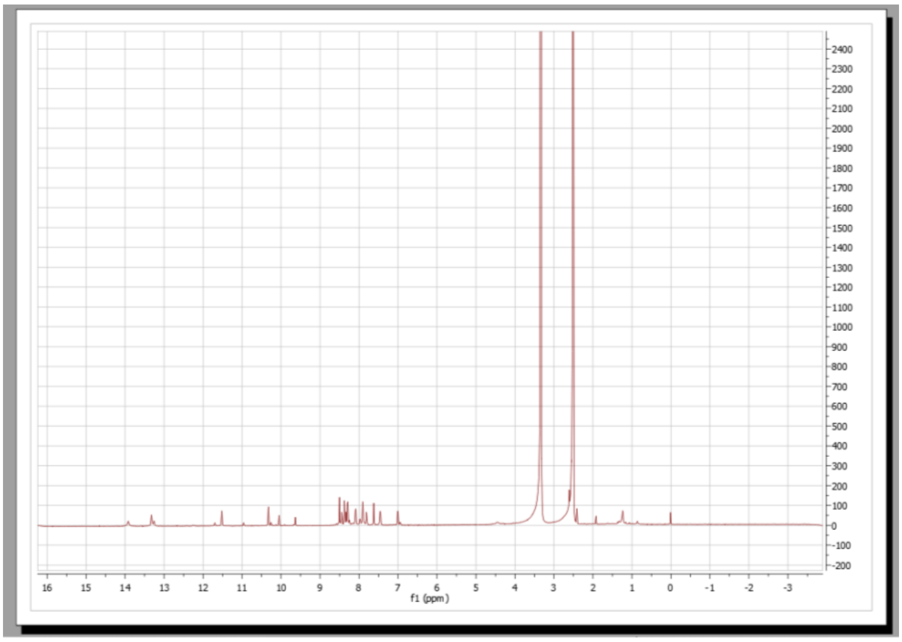

Compound 6:

4-((9H-purin-6-yl)amino)-N'-(5-fluoro-2-oxoindolin-3-ylidene)benzohydrazide (6)

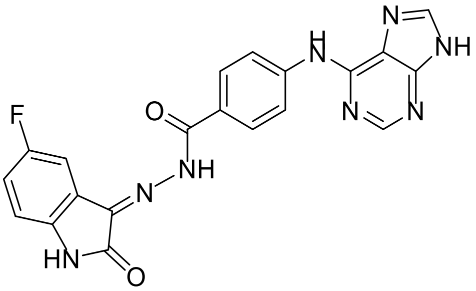

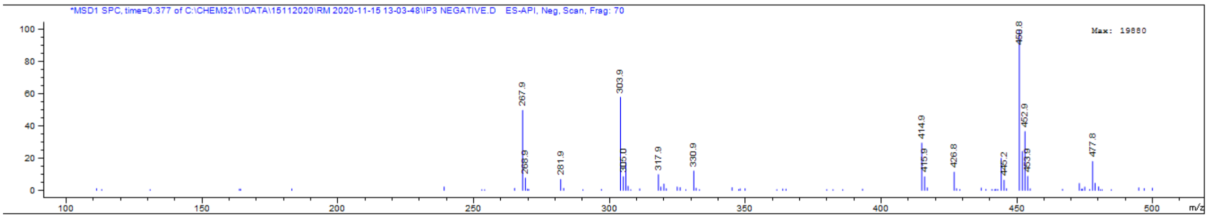

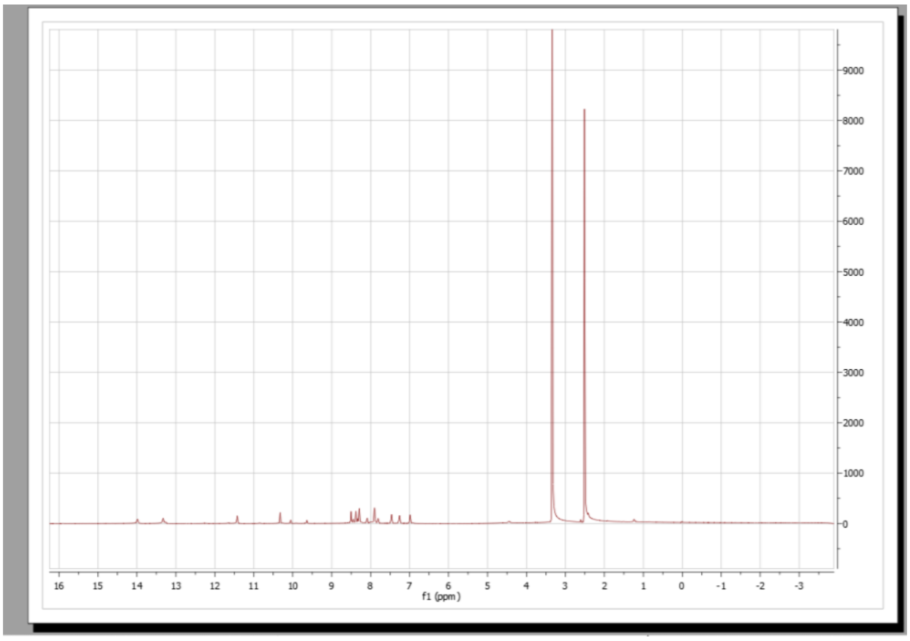

Compound 7:

4-((9H-purin-6-yl)amino)-N'-(5-methyl-2-oxoindolin-3-ylidene)benzohydrazide (7)

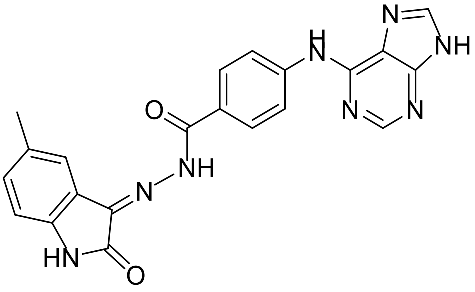

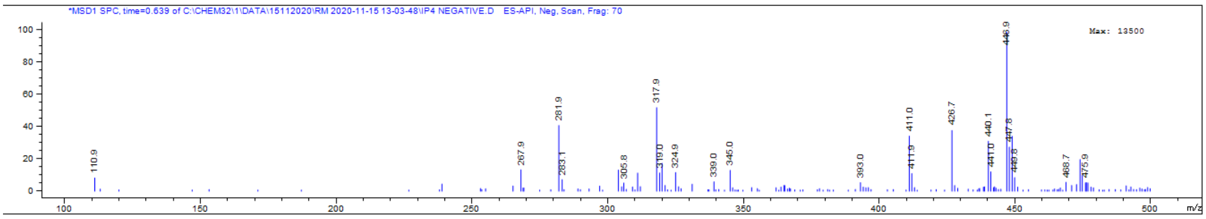

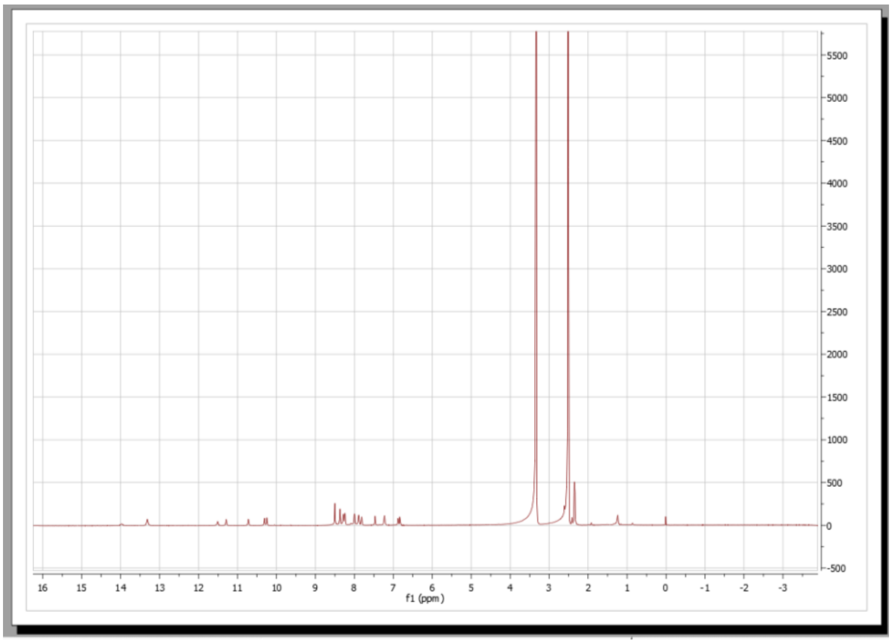

Compound 8:

4-((9H-purin-6-yl)amino)-N'-(5-methoxy-2-oxoindolin-3-ylidene)benzohydrazide (8)

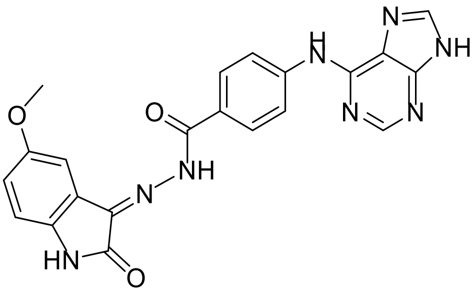

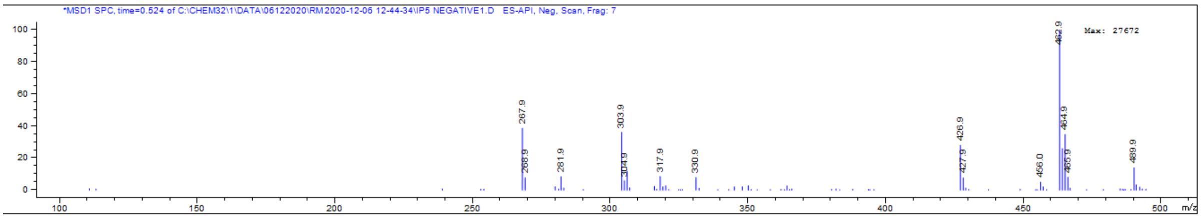

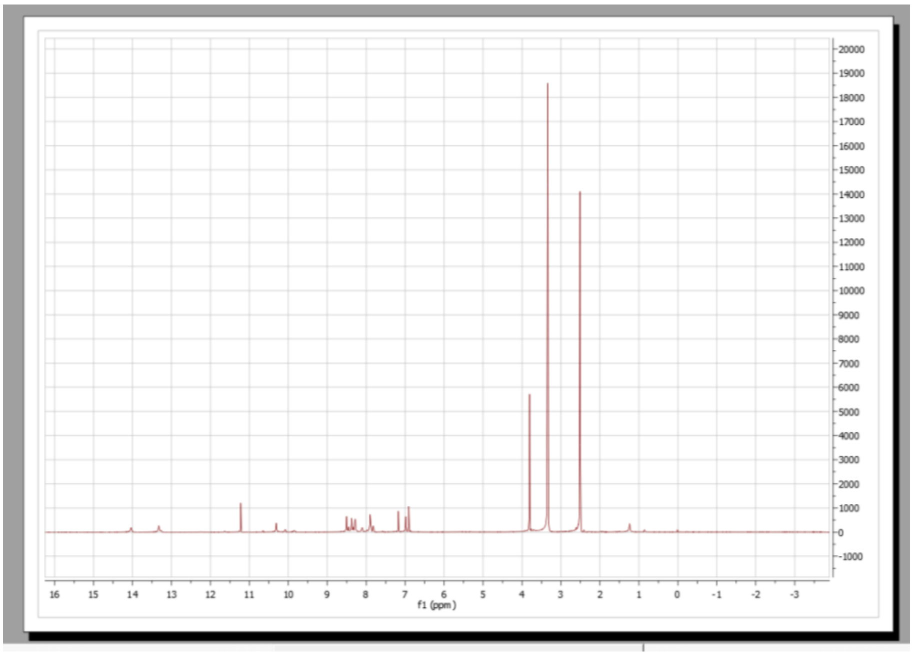

Compound 9:

4-((9H-purin-6-yl)amino)-N'-(5-nitro-2-oxoindolin-3-ylidene)benzohydrazide (9)

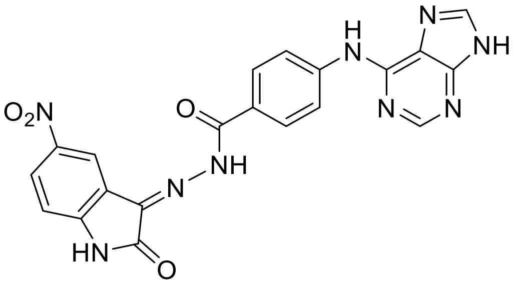

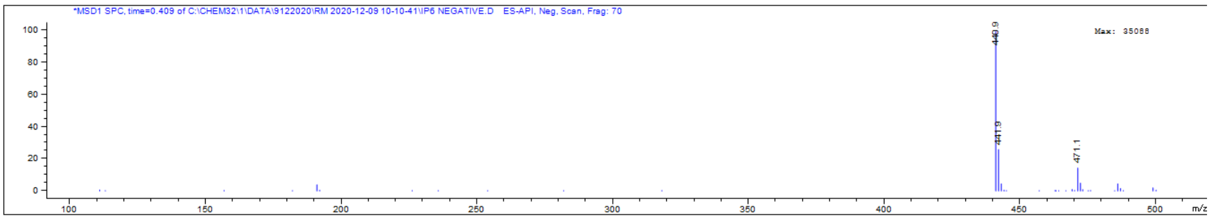

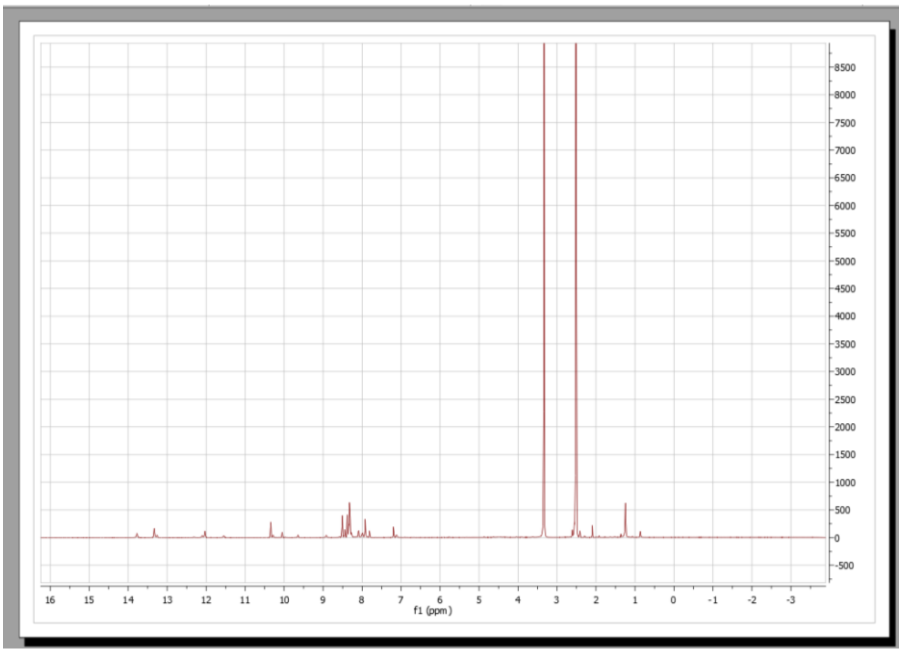

Compound 10:

6-hydrazineyl-9H-purine (10)

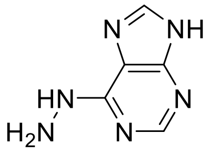

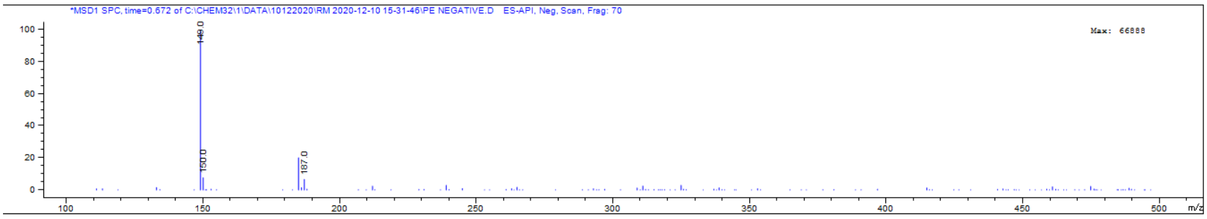

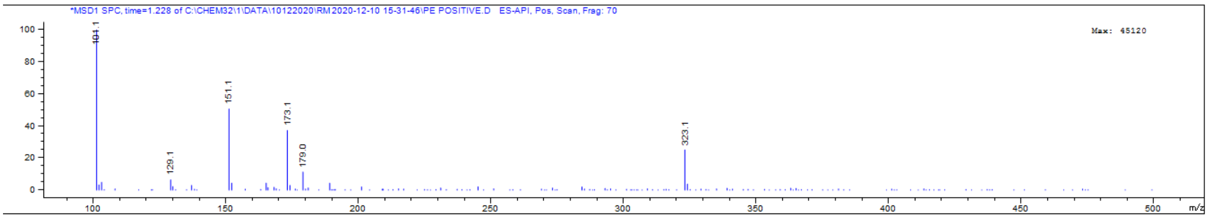

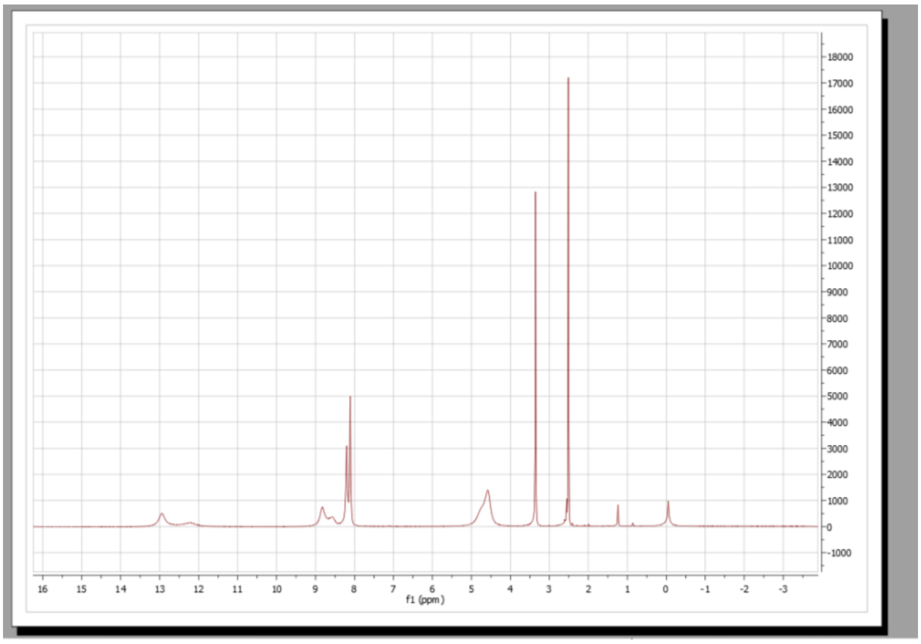

<u>Compound 11:</u>

(E)-3-(2-(9H-purin-6-yl)hydrazineylidene)indolin-2-one (11)

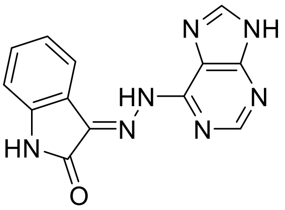

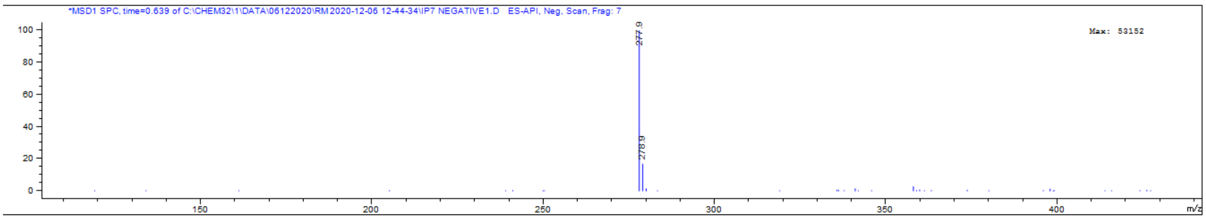

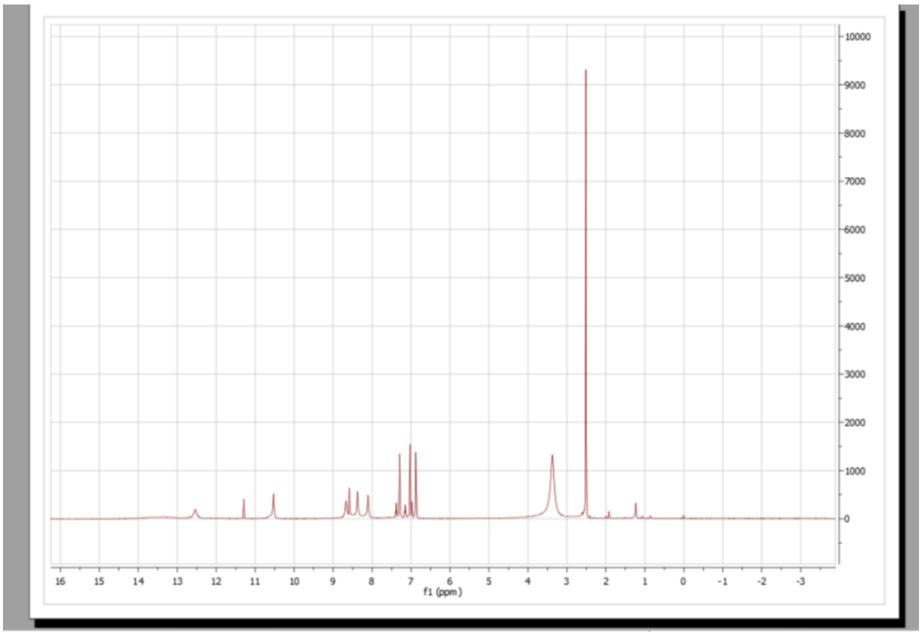

Compound 12:

(E)-3-(2-(9H-purin-6-yl)hydrazineylidene)-5-chloroindolin-2-one (12)

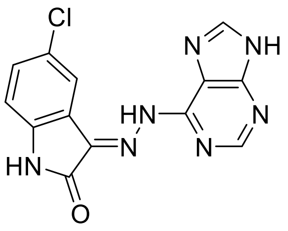

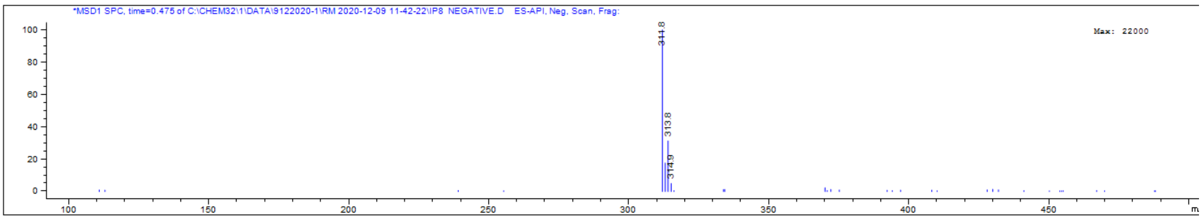

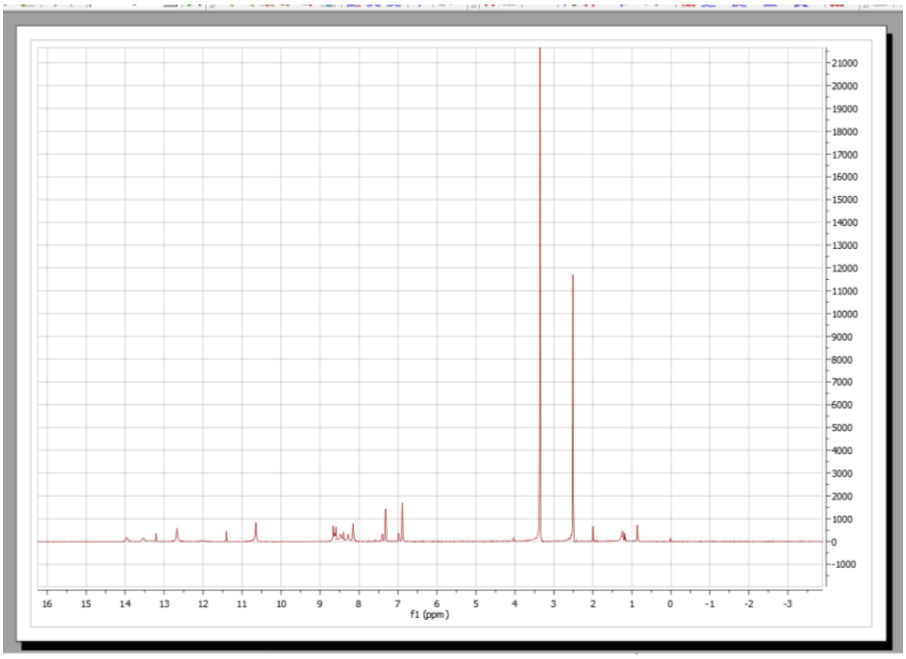

<u>Compound 13:</u>

(E)-3-(2-(9H-purin-6-yl)hydrazineylidene)-5-fluoroindolin-2-one (13)

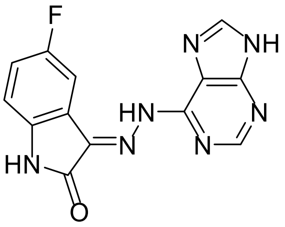

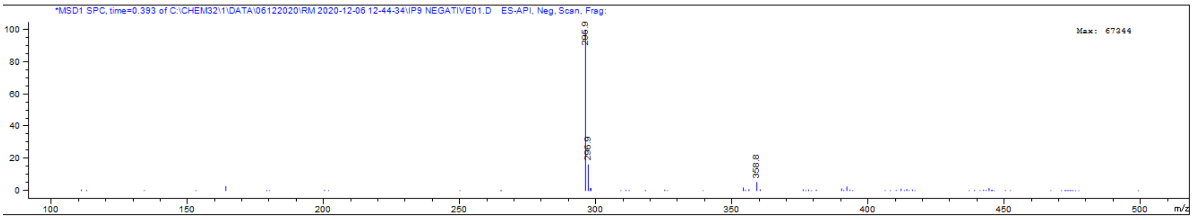

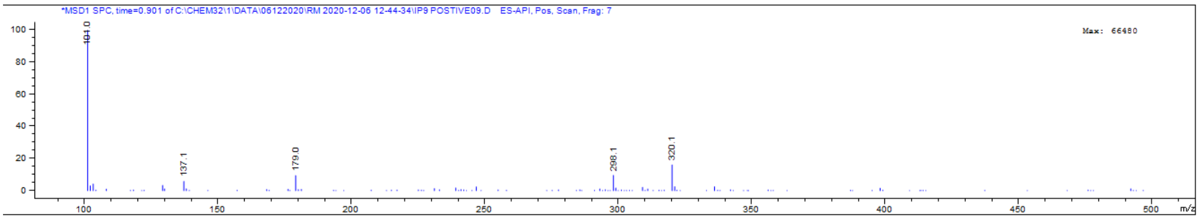

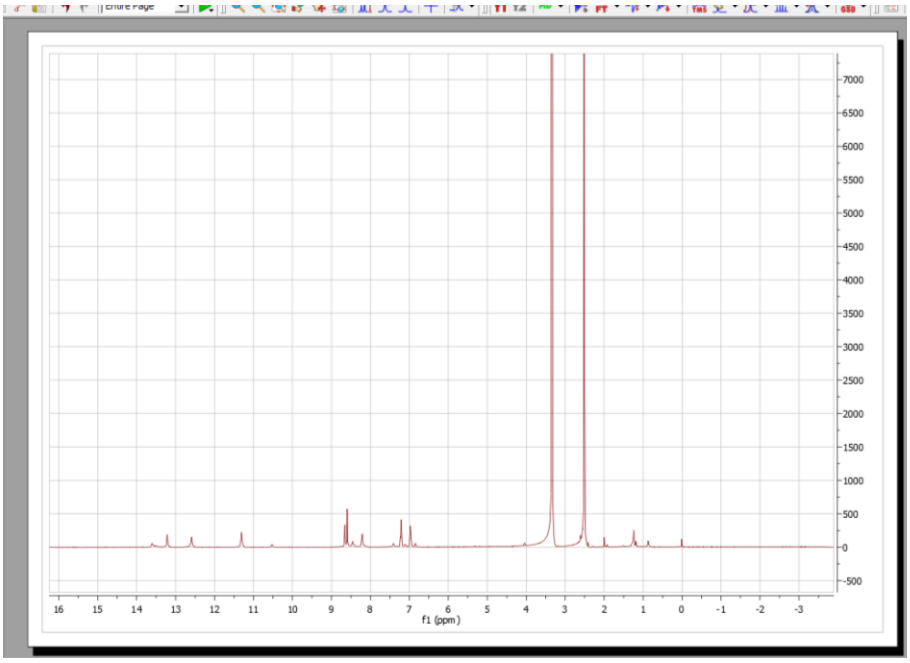

Compound 14:

(E)-3-(2-(9H-purin-6-yl)hydrazineylidene)-5-methylindolin-2-one (14)

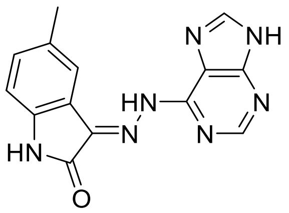

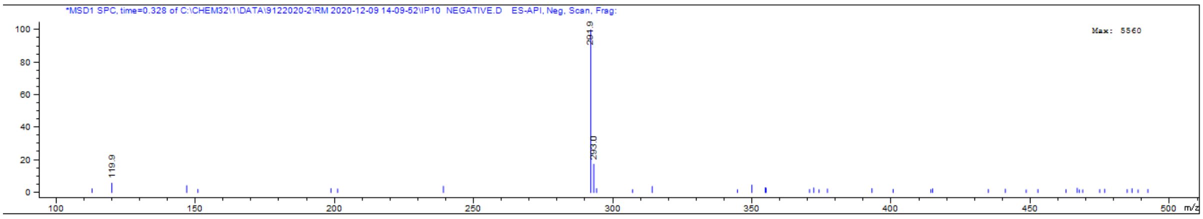

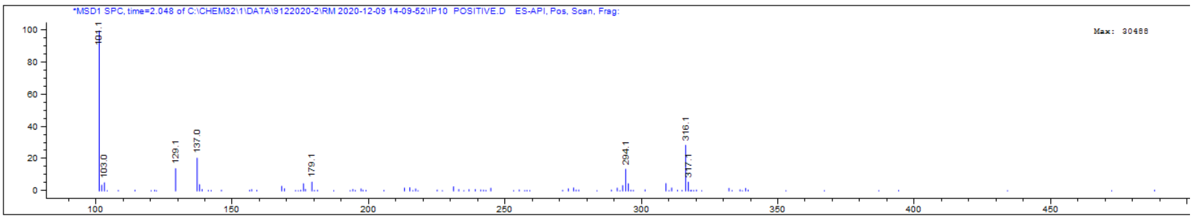

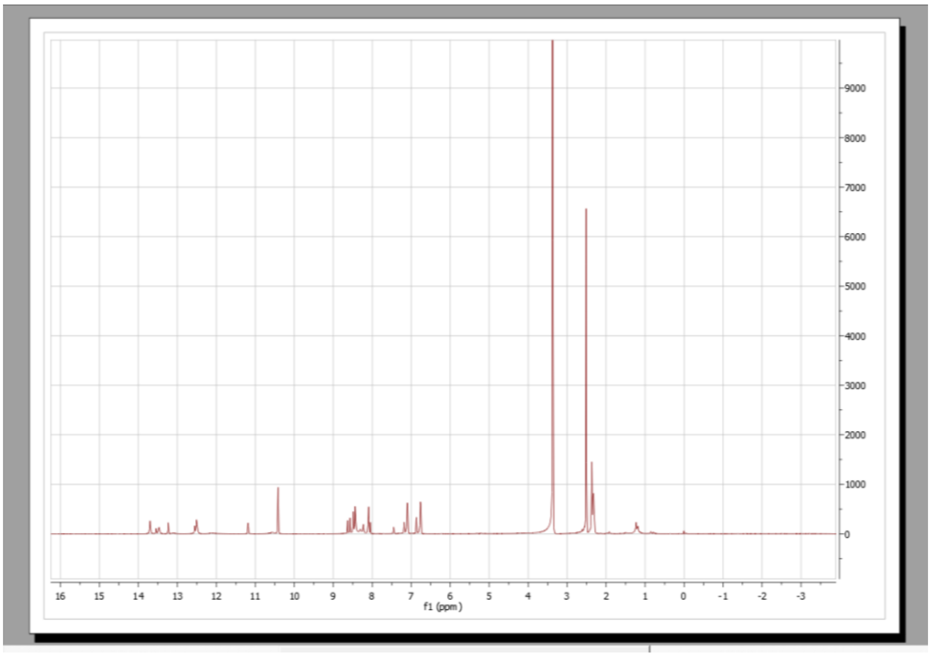

Compound 15:

(E)-3-(2-(9H-purin-6-yl)hydrazineylidene)-5-methoxyindolin-2-one (15)

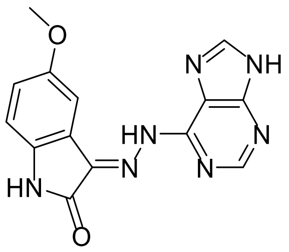

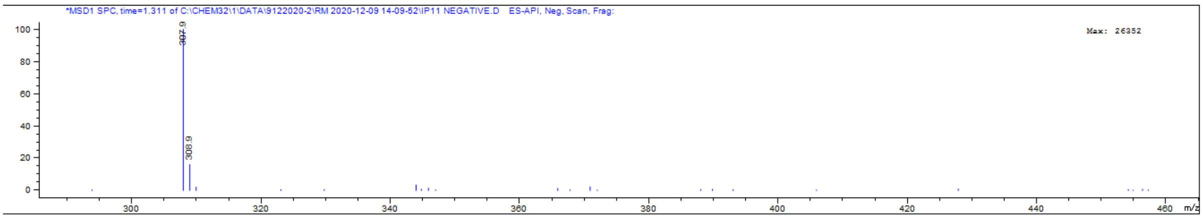

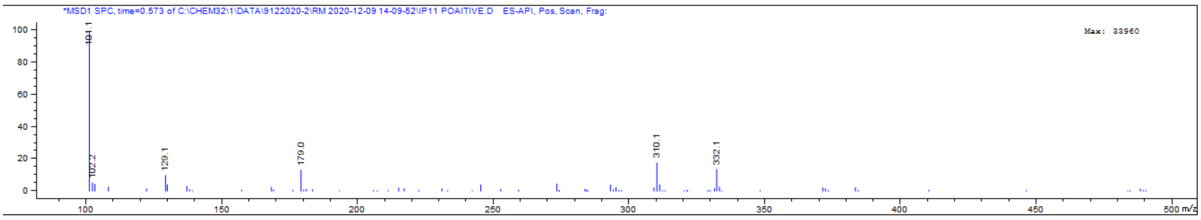

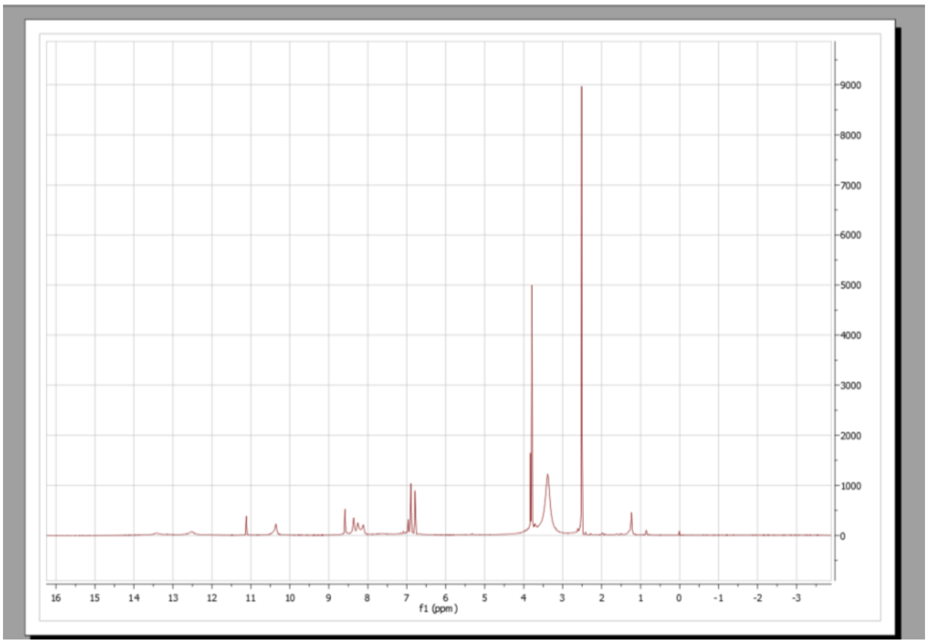

Compound 16:

(E)-3-(2-(9H-purin-6-yl)hydrazineylidene)-5-nitroindolin-2-one (16)

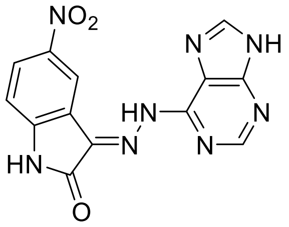
NO2
NH
N
HN
N
N
N
HN
O

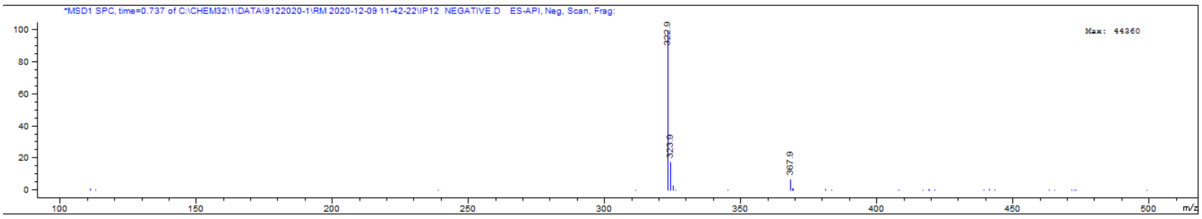
*MSD1 SPC, time=0.737 of C:\CHEM32\1\DATA\9122020-1\RM 2020-12-09 11-42-22\IP12 NEGATIVE.D ES-API, Neg, Scan, Frag:
Max: 44360
322.9
323.9
367.9
m/z

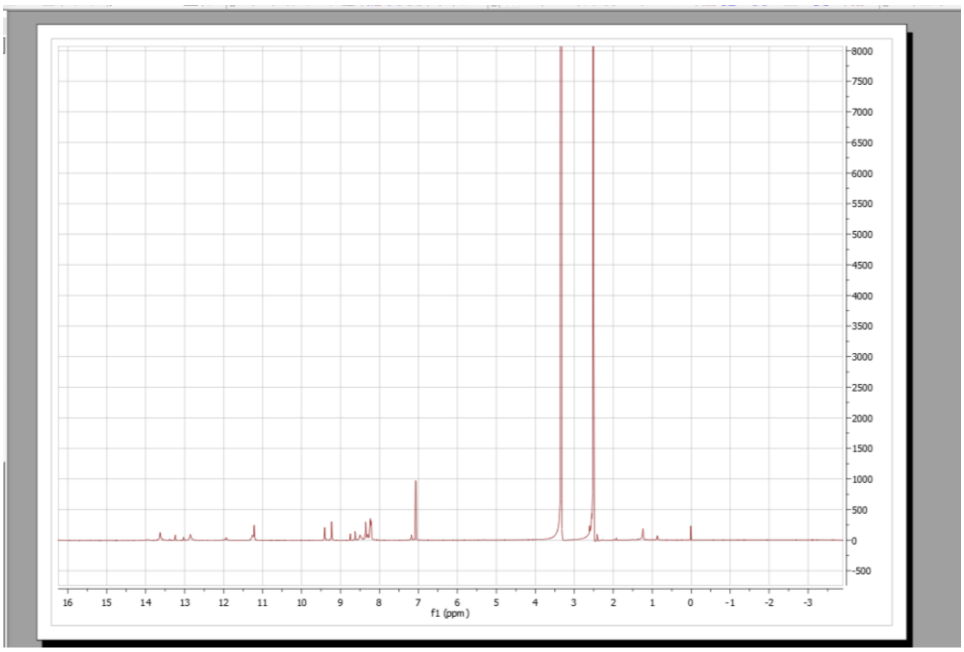
f1 (ppm)
